# ATRX proximal protein associations boast roles beyond histone deposition

**DOI:** 10.1371/journal.pgen.1009909

**Published:** 2021-11-15

**Authors:** William A. Scott, Erum Z. Dhanji, Boris J. A. Dyakov, Ema S. Dreseris, Jonathon S. Asa, Laura J. Grange, Mila Mirceta, Christopher E. Pearson, Grant S. Stewart, Anne-Claude Gingras, Eric I. Campos

**Affiliations:** 1 Genetics & Genome Biology program, The Hospital for Sick Children, Toronto, ON, Canada; 2 Department of Molecular Genetics, University of Toronto, Toronto, ON, Canada; 3 Lunenfeld-Tanenbaum Research Institute, Mount Sinai Hospital, Sinai Health System, Toronto, ON, Canada; 4 Institute of Cancer and Genomic Sciences, University of Birmingham, Birmingham, United Kingdom; Albert Einstein College of Medicine, UNITED STATES

## Abstract

The ATRX ATP-dependent chromatin remodelling/helicase protein associates with the DAXX histone chaperone to deposit histone H3.3 over repetitive DNA regions. Because ATRX-protein interactions impart functions, such as histone deposition, we used proximity-dependent biotinylation (BioID) to identify proximal associations for ATRX. The proteomic screen captured known interactors, such as DAXX, NBS1, and PML, but also identified a range of new associating proteins. To gauge the scope of their roles, we examined three novel ATRX-associating proteins that likely differed in function, and for which little data were available. We found CCDC71 to associate with ATRX, but also HP1 and NAP1, suggesting a role in chromatin maintenance. Contrastingly, FAM207A associated with proteins involved in ribosome biosynthesis and localized to the nucleolus. ATRX proximal associations with the SLF2 DNA damage response factor help inhibit telomere exchanges. We further screened for the proteomic changes at telomeres when ATRX, SLF2, or both proteins were deleted. The loss caused important changes in the abundance of chromatin remodelling, DNA replication, and DNA repair factors at telomeres. Interestingly, several of these have previously been implicated in alternative lengthening of telomeres. Altogether, this study expands the repertoire of ATRX-associating proteins and functions.

## Author summary

ATRX is a protein that is needed to keep repetitive DNA regions organized. It does so in part by binding the DAXX histone chaperone to deposit histone proteins on DNA and assemble structures known as nucleosomes. While important, ATRX has additional functions that remain understudied. To better understand its various biological roles, we first identified the other proteins that are found in its proximity. ATRX-associating proteins were implicated in a range of functions, in addition to histone deposition. Our results suggest that ATRX-associating proteins likely help compact DNA after it is assembled into nucleosomes, and also promote its stability.

We then examined the effect of ATRX on telomeres (repetitive DNA regions at the end of chromosomes). ATRX and at least one of its associating proteins suppressed spurious DNA exchanges at telomeres. To understand why, we then identified proteomic changes that occur at telomeres when ATRX was deleted. Loss of ATRX altered the enrichment of a surprising number of proteins at telomeres, including several DNA damage response and chromatin remodelling proteins.

## Introduction

The alpha thalassemia/intellectual disability, X-linked (ATRX) protein is an SNF2-type ATP-dependent chromatin remodeller/helicase that maintains chromatin over repetitive DNA regions, such as pericentric heterochromatin and telomeres. The protein promotes chromatin compaction [1–3] and prompt DNA damage repair [4, 5]. ATRX deregulation is intimately linked to disease. Germline *ATRX* mutations cause ATR-X Syndrome, a rare X-linked disorder characterized by alpha thalassemia and intellectual disability [6]. Loss of functional ATRX is also frequently observed in cancers that utilize alternative lengthening of telomeres (ALT) to extend telomeres and evade replicative senescence [7, 8]. Moreover, ectopic ATRX expression in ALT+, ATRX-null cells sharply suppresses ALT activity [9].

ALT occurs in 4–15% of cancers, though much higher rates are observed in malignancies of mesenchymal and neuroepithelial origin [10]. ALT is characterized by long and heterogeneous telomeres [11] that are typically associated with a loss of chromatin compaction [12]; presence of telomeric DNA in PML bodies (known as ALT-associated PML bodies, or APBs) [13]; high levels of telomere exchange and synthesis [14–16]; and an accumulation of extrachromosomal, circular telomeric DNA [17]. Recent evidence suggests that ALT is an adaptive response caused by a failure to reactivate telomerase, changes in chromatin compaction, and high levels of DNA damage at telomeres [18–20]. Loss of ATRX is likely an early event in ALT [21], and progressive downstream changes on heterochromatin that enable replication stress at telomeres likely select cells that adopt the ALT phenotype [20]. Cells that use this telomere maintenance mechanism then experience ‘self-perpetuating’ replication stress and double-stranded DNA breaks at telomeres, further sustaining the phenotype [22, 23].

ALT can take different routes (i.e., RAD51-dependent or independent) but is akin to break-induced replication and leads to telomere synthesis [18, 19, 24]. This is thought to occur within APBs, where telomeres and DNA repair factors amass [13]. The formation of APBs, and ALT, are influenced by some of these very same DNA repair proteins, including the MRE11-RAD50-NBS1 (MRN) complex [25–27]. The MRN complex initiates DNA end resection for homology-directed repair [28] and its deregulation promotes ALT activity in some cancer cells [9, 29]. Affinity purification-mass spectrometry (AP-MS) experiments found ATRX to co-purify with the MRN repair complex [30, 31]. ATRX has been proposed to sequester the complex away from telomeres, thereby limiting homology-directed repair associated with ALT [9].

ATRX binds several other proteins. It associates with heterochromatin through its ATRX-DNMT3-DNMT3L (ADD) domain that binds trimethylated lysine 9 on histone H3 (H3K9me3) in the absence of H3K4 modifications [32–34]. A PxVxL-like motif also binds heterochromatin protein 1 (HP1) and influences the localization of the ATRX protein [34, 35]. ATRX forms a complex with the histone chaperone Death Domain-associated Protein 6 (DAXX) to deposit the replication-independent histone variant H3.3 [36, 37], thereby maintaining nucleosome density and genomic integrity over repetitive DNA elements [1–3, 38, 39]. ATRX also influences sister chromatid cohesion at telomeres. It antagonizes macroH2A1.1 deposition, whose accumulation on chromatin impedes tankyrase 1-dependent resolution of sister telomere cohesion when entering mitosis [40]. As a result, cells that lack ATRX have high levels of intra-chromosomal telomeric-sister chromatid exchanges (t-SCEs). In interphase, ATRX promotes sister chromatid cohesion and cells lacking ATRX have high levels of inter-chromosomal telomere exchanges [15].

ATRX clearly plays multiple roles and is influenced by its protein-protein interactions. Comprehensive biochemical characterizations of ATRX protein-protein interactions have been invaluable, but are limited to a few stable interactions (i.e., DAXX, the MRN complex) that remain associated after harsh extractions [30, 31, 41]. We therefore screened ATRX protein-protein associations using proximity-dependent biotinylation (BioID) [42, 43], which tags proteins (prey) that are proximal to a protein of interest (bait) in live cells. Our experiments confirmed robust associations between ATRX and DAXX, as well as with the MRN complex. In addition to established interactions, we also identified uncharacterized proximal associations with proteins involved in rRNA processing, chromatin remodelling, and the DNA damage response. We then examined the role of three poorly characterized proteins that likely differed in function. Our proteomic analyses ascribed roles in ribosome biogenesis, chromatin compaction, and telomere stability for FAM207A, CCDC71, and SLF2, respectively, underscoring the diversity of the cellular roles played by ATRX.

SLF2 (SMC5/6 localization factor 2) was previously identified in a proteomic screen for factors that accumulate at DNA crosslinks, along with SLF1. Together these proteins link the RAD18 DNA damage response protein and the cohesin-like structural maintenance of chromosome 5 and 6 (SMC5/6) complex [44]. SMC5/6 are conserved proteins that facilitate DNA repair, replication, and mitotic segregation, including over repetitive DNA regions [45, 46]. Recent work using magnetic tweezers showed that SMC5/6 captures and compacts DNA tertiary structures in an ATP-dependent manner [47, 48]. In the context of ALT, deregulation of SMC5/6, and E3 SUMO ligase activity conferred by an accessory protein, facilitates APB formation, homology-directed repair, and ALT [49, 50]. We found ATRX to slightly increase the amount of SLF2 on telomeres. While a loss of either ATRX or SLF2 had a limited effect on telomere exchanges, the loss of both proteins showed aberrant telomere exchanges. This indicates a role for these proteins in suppressing telomere recombination. To better understand how ATRX and SLF2 impact telomeres, we performed another BioID screen on the RAP1 telomeric (shelterin complex) protein. A loss of ATRX, SLF2, or both proteins revealed important changes in the binding of chromatin remodelling, DNA replication, and repair proteins at telomeres. Taken together, our data highlight a diverse array of functions by which ATRX influences chromatin and telomeres.

## Results

### BioID reveals novel and diverse associations with ATRX

**Fig 1:**
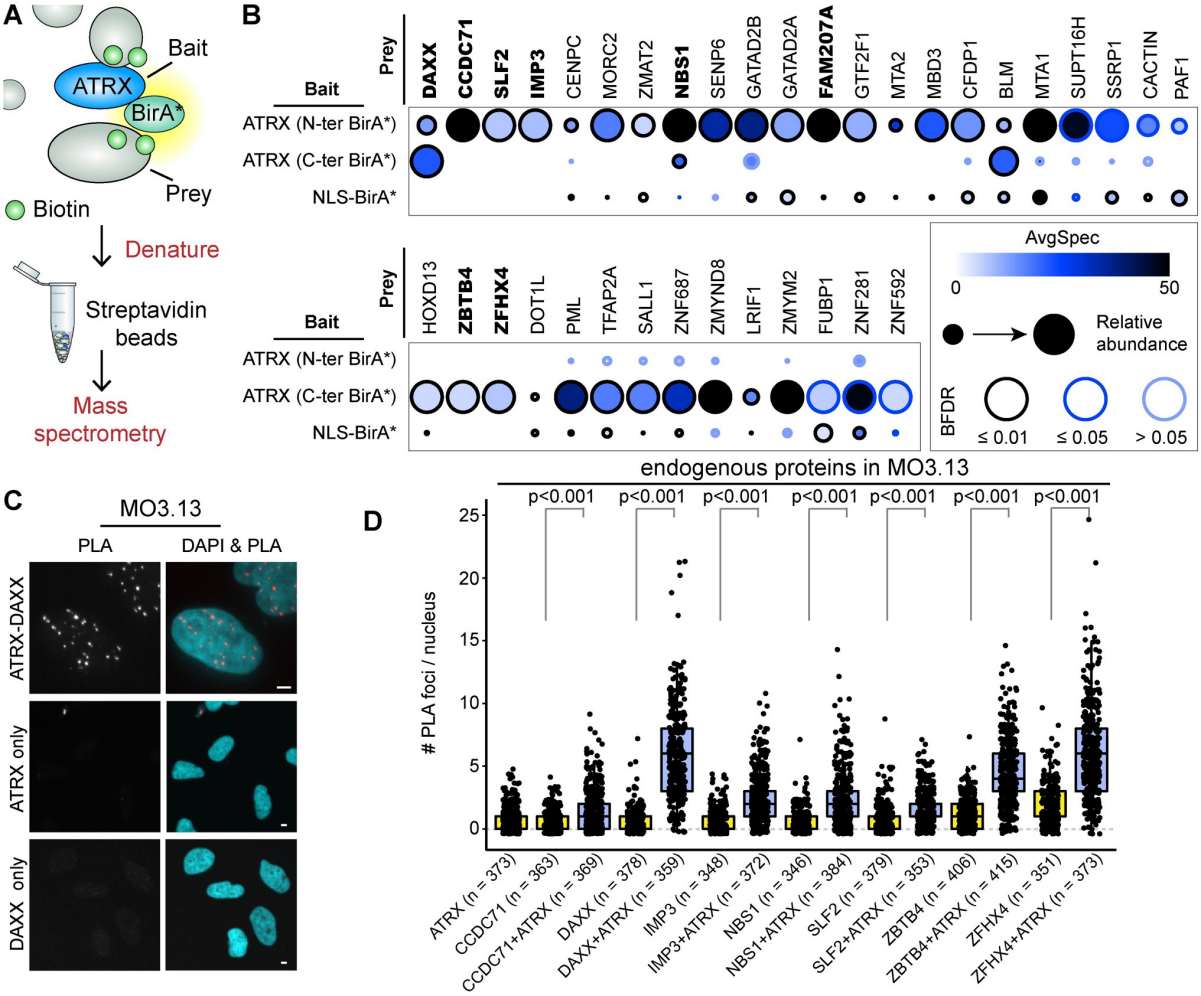
Proximity-dependent biotin identification (BioID) of ATRX-associating proteins. A: Experimental pipeline. The BirA* biotin ligase was fused to ATRX to biotinylate proximal proteins. Labeled proteins were captured on streptavidin beads and analyzed by mass spectrometry. B: Dot plot showing prey proteins identified with ATRX-BirA* that were enriched over endogenous biotinylation (untransfected) and unspecific pan-cellular biotinylation (BirA* alone—BFDR ≤ 5%, SAINT [53]). Data represent two biological replicates. Proteins in boldface remained statistically enriched when the nuclear localization signal (NLS)-BirA* control was used to further filter the ATRX BioID data. C-D: Proximity-ligation assay (PLA) showing proximal associations between endogenous proteins in the MO3.13 human glial cell model. PLA data plots account for ∼100 nuclei in three independent experiments, with the exact number of nuclei assessed indicated in brackets. The p-values were obtained using a 2-sided Student’s t-test with unequal variance. Scale bars = 4 μm.

We theorized that a proximity-based protein labeling methodology in live cells would capture ATRX protein-protein associations that are not represented when using biochemical means. We therefore made use of proximity-dependent biotinylation (BioID) [42, 43] to identify proteins that directly or indirectly associate with the ATRX protein (Fig 1A). ATRX was fused to a FLAG-tagged BirA* biotin ligase at either the N- or C-terminus, and expressed from isogenic, tetracycline-inducible HEK293 Flp-In T-REx cells (herein denoted as HEK293 Flp-In). This produced nuclear bait proteins capable of biotinylating associating proteins in living cells, regardless of whether they are direct or indirect, or static or dynamic. Cells were lysed under stringent conditions, and the proteins purified using streptavidin beads (S1(A) –S1(C) Fig). To identify ATRX-associating proteins, we rigorously filtered the ATRX BioID against a total of 20 negative controls consisting of untransfected cells and isogenic HEK293 Flp-In cells expressing BirA* alone. This identified 36 ATRX proximal associations with a Bayesian false discovery rate (BFDR) ≤ 5% and at least 5 peptides detected across biological duplicates (Fig 1B). BirA* fused to a nuclear localization sequence (NLS) was included for comparison. The DAXX histone chaperone, NBS1 component of the MRN complex, PML protein, BLM helicase, IMP3 U3 snoRNP protein, and the SUPT16H and SSRP1 subunits of the FACT histone chaperone were common to our BioID and published AP-MS experiments in HeLa and HEK293T cells [30, 31, 41, 51], or the curated BioGRID database [52]. Affinity-purified ATRX predominantly captured DAXX, the MRN complex, and the FACT histone chaperone by mass spectrometry [30, 31, 41]. BioID expands this repertoire by considering proximal associations and biochemically labile interactions. Gene ontology analysis of the ATRX BioID showed an enrichment of proteins relating to chromosome organization, chromatin remodelling, and transcriptional regulation (S1(D) Fig).

To identify the most robust associations, we further filtered our ATRX-associating proteins against proteins identified with the NLS-BirA* dataset (Fig 1B and S1 Data). CCDC71, DAXX, FAM207A, IMP3, NBS1, SLF2, ZBTB4, and ZFHX4 remained enriched over all three negative controls (untransfected, BirA* alone, NLS-BirA*). Subcellular fractionation experiments were performed, and while some tubulin was detected in the nuclear fraction, the ATRX-associating proteins that we probed for were all present in the nucleus (S1(E) Fig). To visualize the endogenous protein associations, a proximity-ligation assay (PLA [53]) was performed. PLA generates a fluorescent signal when antibodies that recognize associating proteins are in close proximity to one another (Fig 1C and 1D and S1(F)–S1(I) Fig). Endogenous proteins were followed in the human oligodendrocytic MO3.13 cell model [54] because ATRX is highly expressed in brain tissue and is often deregulated in brain tumours. PLA experiments probing for any one protein alone served as a control. PLA signals were obtained for all but one of the eight proteins listed above. Endogenous FAM207A was excluded from our endogenous PLA analyses because commercial antibodies did not recognize the protein by western blotting.

The endogenous PLA signals for CCDC71 and SLF2 in MO3.13 cells were done using the same antibodies previously used by the Human Protein Atlas [55], but we found these two antibodies also recognized additional proteins by western blotting. The endogenous PLA signals likely reflect true associations, because proteins that cross-react with the antibodies would also need to be in close proximity to generate a signal. Nevertheless, to further ascertain these associations, PLA signals were further obtained between endogenous ATRX and tagged, exogenous CCDC71, FAM207A, and SLF2 (S1(G)–S1(I) Fig). The FLAG tag increased the abundance of PLA background signals, but stark differences in signal intensity were observed when protein associations occurred. Because we lacked PLA data for the endogenous FAM207A protein, we further validated this interaction *in vitro*. Recombinant FAM207A was expressed using a coupled *in vitro* transcription/translation reaction in the presence of biotinylated lysine. FAM207A was immobilized on streptavidin beads, washed, and incubated with recombinant ATRX expressed in SF9 insect cells. Further washing of the beads showed that the two proteins bind one another (S1(J) Fig).

Altogether, the data show that ATRX forms several uncharacterized associations with a diverse group of proteins. To gauge the breadth of functions imparted by ATRX proximal associations, we overviewed three novel ATRX-associating partners for whom there is limited information namely, FAM207A, CCDC71, and SLF2.

### FAM207A is associated with ribosome biogenesis

**Fig 2:**
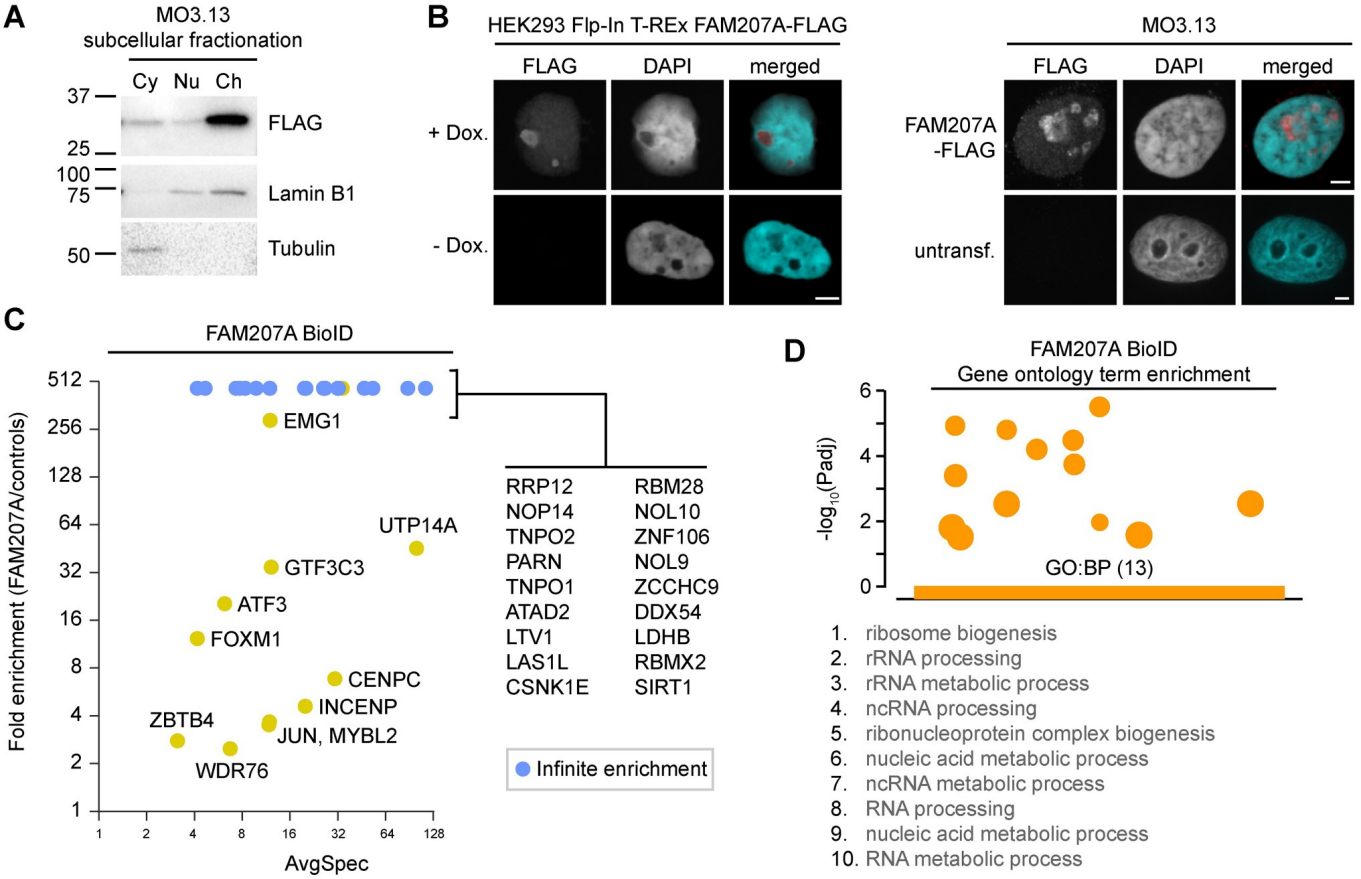
FAM207A is a nucleolar protein involved in ribosome biogenesis. A: Subcellular fractionation of MO3.13 cells expressing FLAG-FAM207A. B: Immunolabeling of exogenous FAM207A in HEK293 Flp-In and MO3.13 cells. C: Fold enrichment of FAM207A BioID prey (BFDR ≤ 5%, SAINT [103]) over negative controls. Data were obtained from biological duplicates. D: Gene ontology terms represented in the FAM207A BioID. Cy—cytoplasm; Nu—nucleus; Ch—chromatin. Scale bars = 4 μm.

An estimated 273 genes orthologous to *FAM207A* (*SLX9*) exist across 256 metazoan species [56]. Subcellular fractionation of MO3.13 and HEK293 Flp-In cells expressing the exogenous protein shows varying levels of FAM207A across the fractions of the two lineages (Fig 2A and S2(A) Fig). This may be due to the exogenous protein expression, but possibly also reflects tissue-specific differences. Immunolabeling of detergent-extracted cells showed that FAM207A strongly localized to the cell nucleoli (Fig 2B). To further ascertain this, cells were then co-labeled for FLAG-FAM207A and IMP3, which is not only an ATRX-associating protein (Fig 1B), but also an established nucleolar protein [57]. Both proteins perfectly co-localized with one another (S2(C) Fig). Curiously, the ATRX-FAM207A PLA signals were not confined to the nucleolus (S1(H) Fig). Because PLA signals form when there is a protein-protein interaction, the signals outside the nucleolus could suggest role(s) for ATRX-FAM207A distinct from those in ribosome biogenesis. It is also worth noting that the Human Protein Atlas database suggests that the protein resides in the Golgi apparatus [58]. However, despite high spectral counts for the protein in our BioID, we were not able to detect the endogenous protein by western blotting when we used the same antibody, even under low stringency conditions (e.g., no tween, high antibody concentration).

A prior affinity purification of the nucleolar NOC4 protein identified FAM207A as a likely component of pre-ribosomal particles [59] and further work suggests a role in the export of 40S pre-ribosomes [60]. Our FAM207A BioID results confirm an association with proteins involved in ribosome maturation (Fig 2C and 2D and S2(D) Fig). Twenty-nine proteins were enriched in the FAM207A BioID (BFDR ≤ 1%) in comparison to the BirA* and NLS-BirA* controls (Fig 2C). A much larger number of proteins were retained when NLS-BirA* was used as a comparative bait instead of a control (S2(D) Fig and S1 Data). We believe that the large number of associated proteins reflects the protein’s involvement in macromolecular assemblies. While ATRX was detected in the FAM207A BioID with a relatively high average spectral count of 27.5, it was filtered out by the negative controls in the SAINT analysis. This perhaps indicates that ATRX only constitutes a minor proportion of the protein associations made by FAM207A, since we otherwise detected the ATRX-FAM207A association through the ATRX BioID and PLAs. Gene ontology groups relating to ribosome biogenesis and rRNA processing predominated (Fig 2D), as previously suggested [59]. FAM207A, however, also associated with proteins involved in chromosome and transcriptional regulation, again suggesting more than one biological function.

### CCDC71 is a chromatin-bound protein that associates with HP1

**Fig 3:**
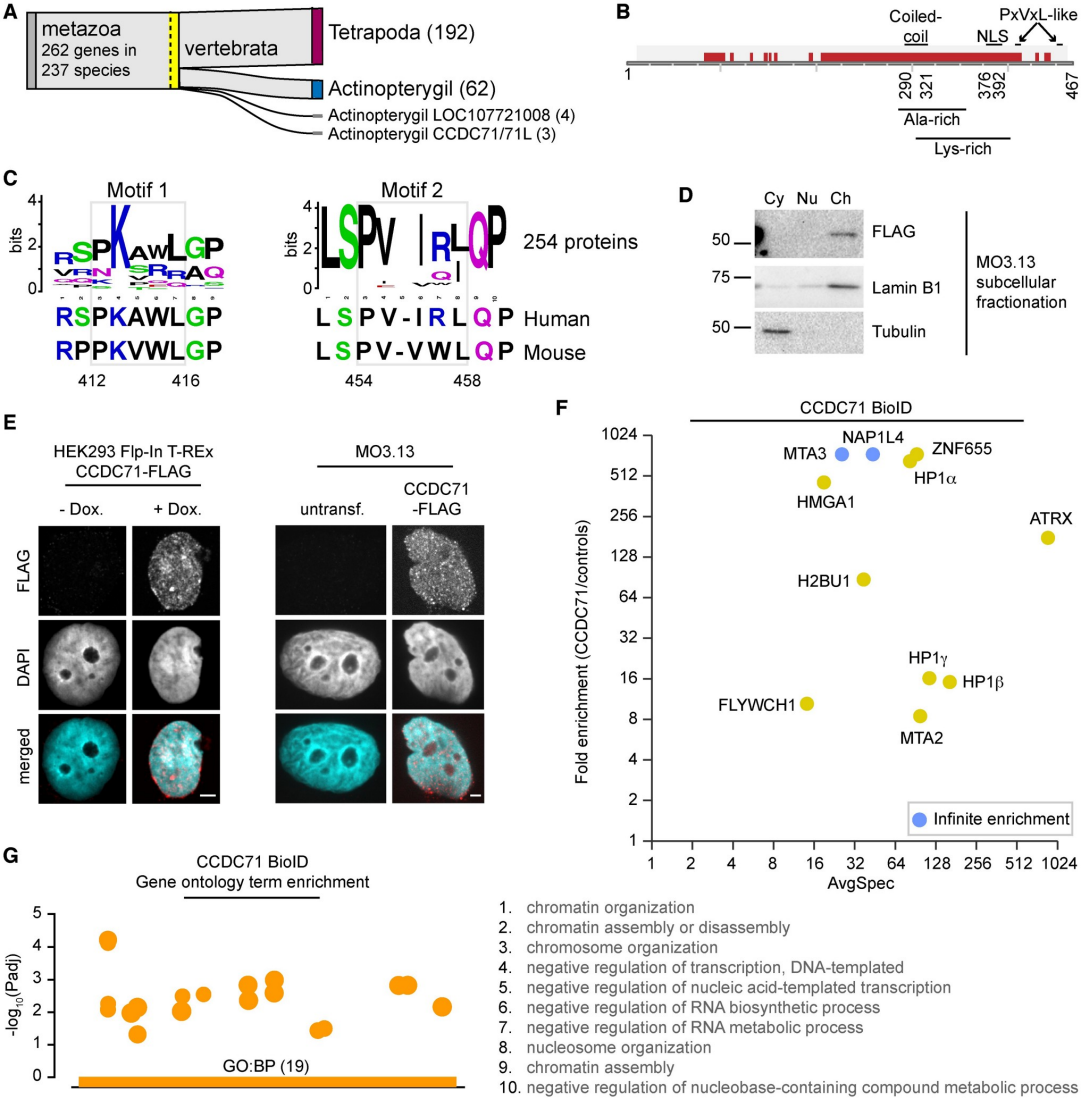
CCDC71 is a chromatin-bound protein that associates with HP1. A: Hierarchical catalogue of orthologs (OrthoDB v10.1) [56] diagram showing 262 putative genes within the *CCDC71/71L* family. B: Graphical depiction of the CCDC71 primary structure and features identified using Pfam [110] and Prosite [111]. Putative PxVxL-like motifs are also shown. The CCDC71/71L domain is shown in light grey and putative disordered regions in dark red. C: Sequence analysis of 254 CCDC71 orthologs aligned around the two PxVxL-like motifs. The sequence alignment was generated using COBALT [112] and visualized using WebLogo [113]. D: Subcellular fractionation of MO3.13 cells expressing FLAG-CCDC71. E: Immunolabeling of exogenous CCDC71 in HEK293 Flp-In and MO3.13 cells. F: Fold enrichment of CCDC71 BioID prey (BFDR ≤ 5%, SAINT [103]) over negative controls. Data were obtained from biological duplicates. G: Gene ontology terms represented in the CCDC71 BioID. Scale bars = 4 μm.

Current databases and our own experimental data implicate CCDC71 in chromatin organization. CCDC71 belongs to the coiled-coil domain 71/71L family found within vertebrates (Fig 3A). In addition to a coiled-coil region, the protein harbours an NLS and several regions that are predicted to be disordered (Fig 3B). The mouse protein contains two canonical PxVxL sequences, a motif found in proteins that bind HP1 [35]. Variation within the sequence is tolerated to various degrees [35, 61], and both the human and the consensus sequence from 254 putative orthologs contain PxVxL-like sequences at these two locations (Fig 3C). A canonical PxVxL sequence is also found in the human CCDC71L protein, a shorter family member that shares a high degree of identity within the N-terminal half of CCDC71 and its C-terminal extremity (S3(A) Fig). It was, therefore, not surprising to see that the exogenous protein was almost exclusively found in the chromatin fraction of MO3.13 and HEK293 Flp-In cells (Fig 3D and S3(B) Fig).

To our knowledge, there are no reports on CCDC71, but data gathered by the Human Protein Atlas [55] suggest that the protein locates to the nuclear periphery in HeLa cells, with some cell types showing a more nuclear signal. Immunolabeling in HEK293 Flp-In cells showed pan-nuclear granular signals (Fig 3E), a pattern also seen in the ATRX-CCDC71 PLA experiments in HEK293 Flp-In cells (S1(G) Fig). Putative CCDC71 protein-protein interactions reported in BioGrid [52] suggest a role in chromatin compaction. They include histone H3.3, the EED subunit of the polycomb repressive complex 2 (PRC2), the RbAp46 histone chaperone, and the HDAC1 histone deacetylase. We could indeed co-immunoprecipitate H3.3, EZH2 (the catalytic subunit of PRC2), and HDAC1 with CCDC71 (S3(C) Fig). To obtain a clearer portrait of CCDC71 functions and its proximal protein associations, a BioID analysis was also performed for this protein. While HDAC1 was represented, the most prevalent associations included ATRX, all three HP1 isoforms, the NAP1 histone chaperone, and components of the NuRD chromatin remodelling protein complex (also represented in the ATRX BioID; Fig 3F, S3(E) Fig and S1 Data). As expected, a gene ontology analysis of the CCDC71 BioID results included terms related to chromosome and chromatin organization (Fig 3G). This shows that ATRX and CCDC71 share important functions in chromatin regulation.

### SLF2 prevents spurious telomere recombination

**Fig 4:**
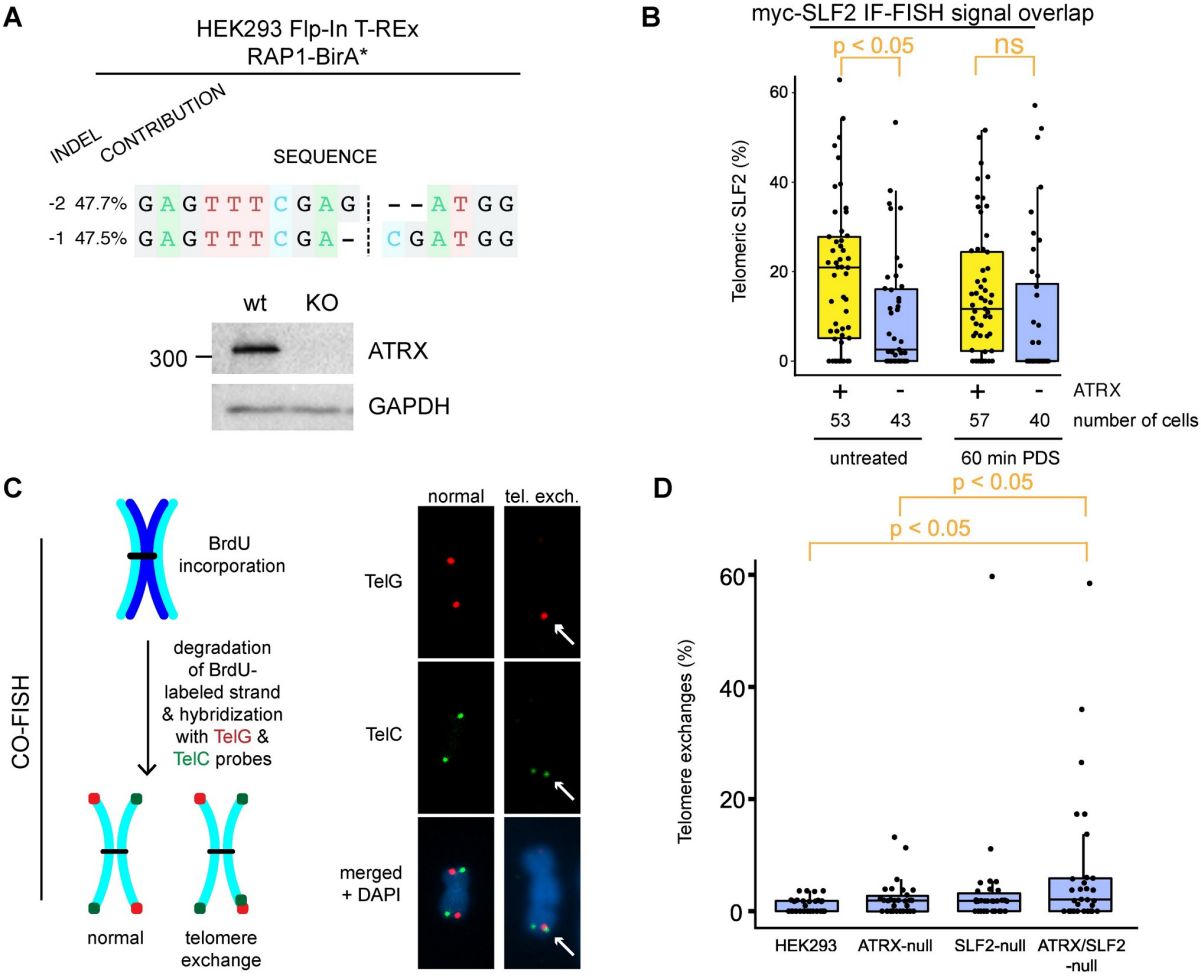
Loss of ATRX and SLF2 enables telomere exchanges. A: DNA sequencing and western blotting confirming the disruption of ATRX expression in CRISPR-Cas9-edited HEK293 Flp-In T-REx cells inducibly expressing RAP1-BirA*. B: Relative signal overlap between immunolabeled SLF2 and telomere fluorescence *in situ* hybridization (IF-FISH), in ATRX-expressing or -null HEK293 Flp-In cells. Cells were exposed to 10 μM pyridostatin (PDS, G4 stabilizer) for 60 min. At least 40 cells per condition were analyzed using a CellProfiler colocalization pipeline [106]. ATRX loss decreased SLF2 recruitment to telomeres in the absence of pyridostatin. C: Schematic of dual-colour chromosome-orientation fluorescence *in situ* hybridization (CO-FISH) [64] using PNA probes to label C- and G-rich telomere strands (left). Examples of normal CO-FISH signals and of a chromosome end with a telomere exchange are shown (right). D: Relative amount of telomere exchanges in ATRX, SLF2, and ATRX/SLF2 KO HEK293 Flp-In cells. At least 30 mitotic spreads per condition were counted and the percent of telomeric exchanges plotted. p-values were obtained using a 2-sided Student’s t-test with unequal variance.

The SLF2 proteome was previously described [44], but the association with ATRX is novel. It was found that SLF1/2 loads the cohesin-like SMC5/6 proteins at sites of DNA damage [44]. A genetic interaction between *ATRX* and *SMC5* and *SMC6* was recently reported [62] and deregulation of SMC5/6 in ALT-positive cells promotes APB formation and telomere exchanges [49]. To determine if ATRX influences SLF1/2 and SMC5/6 recruitment to telomeres, we disrupted ATRX expression in HEK293 Flp-In cells using CRISPR-Cas9 (Fig 4A), and quantified SLF2 occupancy at telomeres. Exogenous SLF2 and endogenous SMC5 were imaged by immunofluorescence and telomeric regions identified using fluorescence *in situ* hybridization (IF-FISH). SLF2 and SMC5 formed foci whose signals strongly overlapped with one another (S4(A) and S4(B) Fig). Most SLF2 and SMC5 foci formed outside telomeres, but approximately 20% of the signals coincided with those from the telomeric FISH probe (Fig 4B). The proportion of SLF2/SMC5 overlap with telomeres decreased in the ATRX-null cells. G-quadruplex (G4) structures can form at telomeres, where they cause DNA damage if not properly resolved. The analysis was therefore repeated in cells exposed to the G4-stabilizing small molecule, pyridostatin [63], but the treatment had no impact on the SLF2/SMC5—telomere signal overlap (Fig 4B).

ATRX expression restrains telomere exchanges [9, 15, 40]. We therefore wanted to determine if ATRX and SLF2 together repress t-SCEs. To investigate this, we disrupted SLF2 in ATRX-expressing and -null HEK293 Flp-In cells (S4(C) and S4(D) Fig), and performed chromosome-orientation fluorescent *in situ* hybridization (CO-FISH) [64] to visualize and quantify telomere exchanges (Fig 4C and 4D). As recently reported [15], we observed increased levels of t-SCEs (dual signal on matching sister chromatid ends), but also a high proportion of exchanges on telomere ends of single chromatids in our ATRX-null cells (Fig 4D). This can indicate non-allelic exchanges and the events were therefore grouped as ‘telomere exchanges,’ as previously done [15]. Low p-values were obtained when comparing telomere exchange rates between the single null cells and the parental HEK293 Flp-In (e.g., 0.059 and 0.15 for ATRX- and SLF2-null cells, respectively), but significance was reached in double ATRX/SLF2-null cells (Fig 4D). This suggests that both proteins inhibit spurious exchanges. However, the lack of a clear synergistic effect may signify that the proteins do so independently of one another. In the ALT-positive (ATRX-null) U2OS cells, an *SLF2* gene disruption again caused a significant increase in telomere exchanges (S4(E) Fig). Altogether, this shows that ATRX has some influence on SLF2 recruitment, and a loss of both proteins permits high levels of telomere exchanges.

### ATRX and SLF2 depletion alters protein enrichment at telomeres

**Fig 5:**
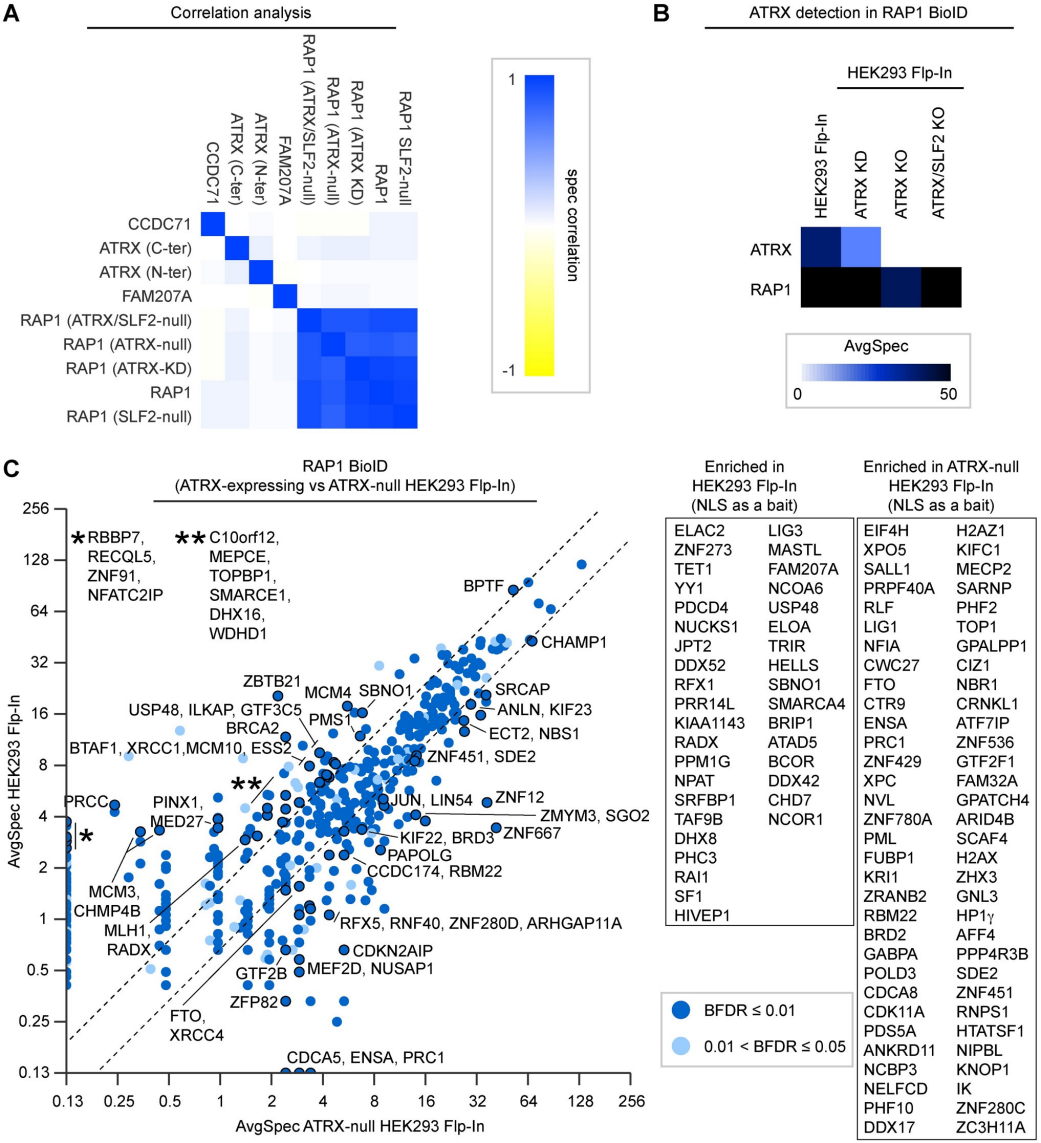
Loss of ATRX changes the enrichment of a subset of proteins at telomeres. A: Correlation between BioID bait proteins. RAP1 BioID experiments strongly correlated with one another independently of ATRX expression. B: Relative ATRX detection by RAP1 BioID in ATRX-expressing and isogenic cells where ATRX expression was knocked-down (KD) using shRNA or knocked-out (KO) using CRISPR-Cas9. C: Fold change of prey proteins identified by RAP1 BioID (BFDR ≤ 5%, SAINT [103]) in ATRX-expressing vs. ATRX-null HEK293 Flp-In cells. Labeled proteins had ≥2.5 average spectra and ≥1.5-fold change between conditions. Proteins labeled in the scatter plot remained significant when NLS-BirA* was used as a control. Inset box represents additional proteins identified with NLS-BirA* as a bait (less stringent).

A loss of ATRX causes gradual changes on telomeres [20], so we wanted to determine if its loss, or that of SLF2, caused immediate observable changes in protein enrichment at telomeres. To examine this, we performed BioID on the RAP1 (TERF2IP) subunit of the shelterin complex, in parental HEK293 Flp-In cells and compared the results to that of the same BioID done in isogenic SLF2-null, ATRX-null, or cells stably expressing ATRX shRNA (Fig 5 and S5 Fig). The same BioID strategy, but using TERF1 as a bait, previously identified changes in the telomeric proteome of ALT-negative and ALT-positive cells [65]. While we did not enrich for all the shelterin components, TERF2 was one of the most abundant prey proteins identified in the RAP1 BioID (S1 Data). Importantly, 38% of the 343 proteins identified by our RAP1 BioID in HEK293 Flp-In cells were also identified by the previously reported TERF1 BioID in HeLa cells [65], and 55% our of preys were also identified by other proteomic techniques used to identify telomeric proteins (namely, PICh [66], QTIP [67], C-BERST [68], and TERF1 BioID [65]; S5(C) Fig). A similar overlap was seen when using NLS-BirA* as an additional control in our BioID, but we first omitted the control from this analysis to identify as many changes as possible (data were still filtered against untransfected cells and BirA*). As expected, RAP1 BioID experiments performed in parental and ATRX- or SLF2-depleted HEK293 Flp-In cells were far more similar to one another than our other BioID experiments with other baits (Fig 5A).

**Fig 6:**
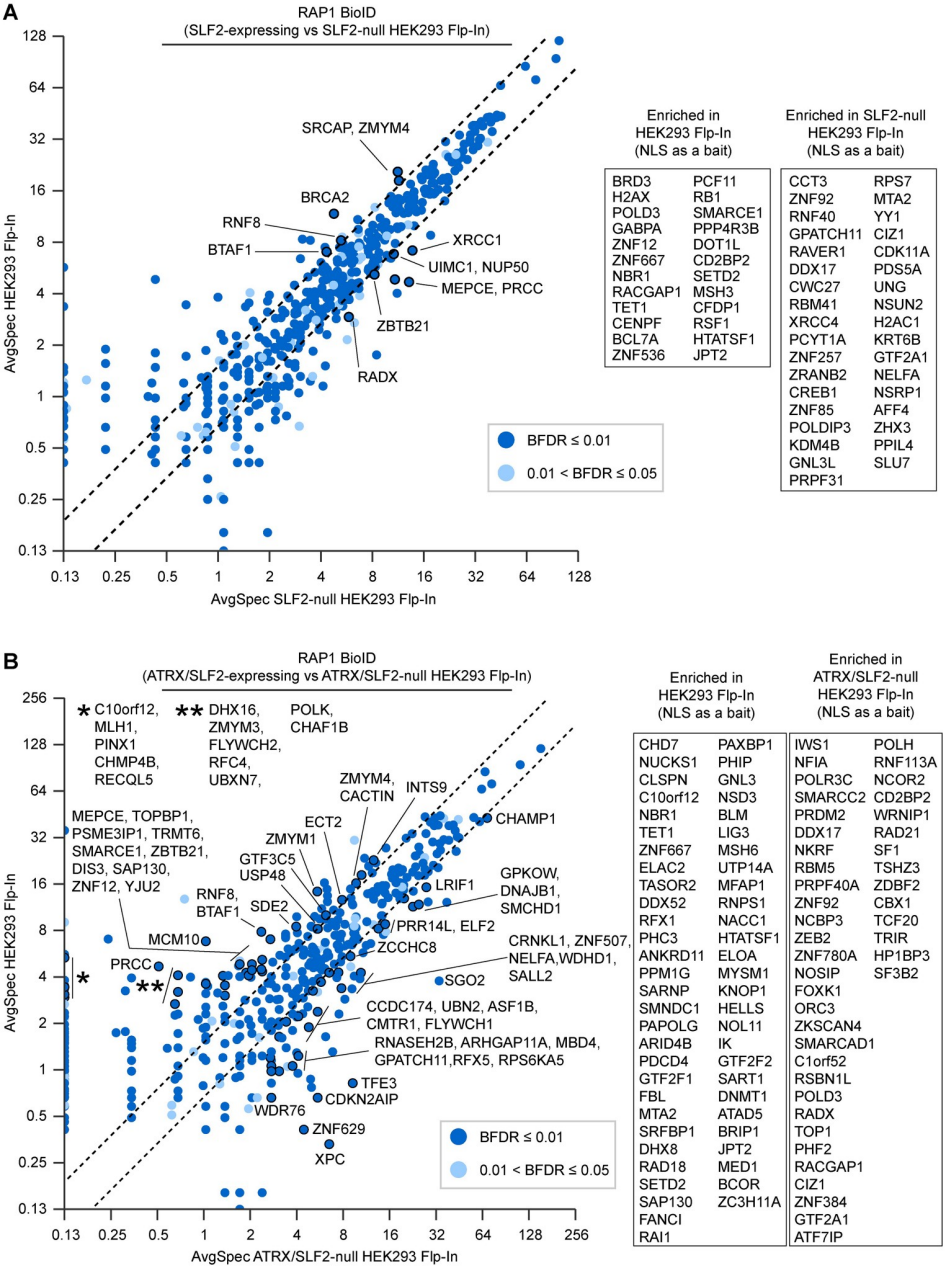
Changes at telomeres caused by a loss of SLF2 and ATRX. Fold change of prey proteins identified by RAP1 BioID (BFDR ≤ 5%, SAINT [103]) in A: SLF2-null, and B: ATRX/SLF2-null vs. ATRX- and SLF2-expressing HEK293 Flp-In cells. Labeled proteins had ≥2.5 average spectra and ≥1.5-fold change between conditions. Proteins labeled in the scatter plot remained significant when NLS-BirA* was used as a control. Inset box represents additional proteins identified with NLS-BirA* as a bait (less stringent).

The ATRX knockout (Fig 5C) and knockdown (S5(D) Fig) caused surprisingly vast changes at telomeres, with proteins involved in chromatin maintenance, DNA replication, and repair showing both gain and loss of abundance. The relative abundance of some the proteins that associated with ATRX (such as NBS1 and PML) also changed in relative abundance at telomeres, but the most prominent changes involved other non-associating proteins. Loss of ATRX therefore carries indirect downstream changes that alter telomeres. We then examined the changes that occur in SLF2-null and isogenic SLF2/ATRX double null cells (Fig 6). In comparison to ATRX, SLF2 depletion had a far more limited effect on telomeric proteins, although chromatin remodelling and DNA repair proteins again changed in abundance at telomeres (Fig 6A). The double-null cells largely recapitulated the effects seen in the individual gene knockouts, suggesting that most of the effects are additive.

The increase in PIN2/TERF1-interacting telomerase inhibitor 1 (PINX1) at telomeres upon SLF2 loss is interesting because the protein inhibits telomerase activity [69] and promotes TERF1 binding to telomeres [70]. Proteomic changes caused by a loss of ATRX and/or SLF2 therefore likely destabilize telomeres.

## Discussion

Isolation of ATRX-binding proteins yields biochemically-stable interactions with DAXX and components of the MRN protein complex [30, 31, 41], yet ATRX binds additional proteins. The discrepancy is likely due to the need for harsh extraction methods to analyze its interactions on chromatin. With the advent of proximity-based labeling techniques [43], we were able to surmount this technical limit and identify 36 ATRX-associating proteins (Fig 1B). Our BioID results are high confidence since the data were stringently filtered against 20 BioID control replicates. To identify the strongest associations (whether direct or indirect), we further filtered the data against an additional 14 biological replicates of NLS-BirA*, where indicated. While DAXX and NBS1 were enriched in the BioID experiments performed with BirA* at either the N- or C-terminus of the protein, there was a surprising degree of variation between the two baits. The differences could be due to spatial constraints within these associations, but also hindrance or misfolding caused by the bulky biotin ligase. However, both protein termini are predicted to be of very low complexity and unstructured [71], making misfolding of BirA*-tagged ATRX less likely. Spatial specificity via BioID has also been reported for other proteins, where data obtained by biotin ligases at the N- and C-terminus of the bait protein were seen as complementary [72, 73]. We were indeed able to visualize the high confidence associations by PLAs regardless of the BirA* position (Fig 1C and 1D), suggesting that the differences are most likely due to spatial constraints on the ∼300 kDa protein. It is interesting to note that ATRX has two DAXX-interacting regions: a weak N-terminal one (spanning a.a. 321–865) and a stronger C-terminal one via ATRX’s DAXX Binding Motif (a.a. 1189–1326) [74] and that our BioID results reflect that difference. The interaction with PML is also mediated by the C-terminal extremity of ATRX [75], again recapitulated in the BioID.

Interestingly, the previous affinity-purified ATRX mass spectrometry experiments, and our ATRX BioID dataset, lacked reported interactions with HP1 [35, 76, 77], macroH2A.1 [78, 79], and MeCP2 [80, 81]. These interactions had been established by immunoprecipitation/western, co-localization, or *in vitro* experiments. While HP1*β* (CBX1), HP1*γ* (CBX3), and macroH2A.1 were detected in the ATRX BioID, they were filtered out because they were also labeled by our negative controls (S1 Data). MeCP2 was, however, only detected by the negative controls. Some of the results could reflect cell type-specific differences and/or spatiotemporal limits of the biotin ligase. Interestingly, HP1 was later captured not by ATRX, but rather a novel ATRX-associating protein (see below). The specific identification of the NBS1 component of the MRN complex (and lack of MRE11 and RAD50 subunits) does not necessarily mean that ATRX only associates with NBS1, but could reflect the relative position of the biotin ligase over the protein complex. Indeed, others showed that ATRX also co-purifies with the other MRN complex subunits [30, 31].

Because the ATRX associations enriched for ribosomal, chromatin, and DNA repair proteins, we then examined three associating proteins for which there is limited information, but that likely reflects these functions. FAM207A, CCDC71, and SLF2 were chosen because there is limited information on the proteins, and associations reported in BioGrid [52] suggested that they had very different biological functions. Our data confirm that FAM207A is predominantly nucleolar (Fig 2B). This is interesting because ATRX does enrich over rDNA repeats across different life kingdoms [76, 82–84]. ATRX depletion in mESCs destabilizes rDNA repeats through a loss of histone deposition and repressive histone marks, and a decrease in rDNA copy numbers and rDNA transcription that renders cells sensitive to RNA polymerase I inhibitors [85]. The FAM207A protein previously co-purified with isolated pre-ribosomal particles and RNAi-mediated depletion of the protein implicated it in 40S precursor maturation [59]. Our data concur and demonstrate that FAM207A associates with proteins involved in ribosome biogenesis (Fig 2C and 2D). However, FAM207A BioID also identified proteins with functions in gene transcription and chromosome organization (S2(D) Fig), and our PLA analysis showed that the ATRX-FAM207A association is not exclusive to the nucleolus (S1(H) Fig). In fact, like ATRX, yeast FAM207A (Slx9) is proposed to bind G-quadruplex DNA structures that form over repetitive G-rich DNA [86] and it will be very interesting to find the exact role of the ATRX-FAM207A association.

A second ribosomal protein, IMP3, was also identified as an ATRX-interacting protein in our BioID analysis (Fig 1B). IMP3 is a conserved nucleolar rRNA processing protein also implicated in ribosome biogenesis [57]. The ATRX-IMP3 interaction was also reported in the BioPlex interactome database [51]. While FAM207A BioID did not capture IMP3, it is possible that their functions converge. It is also worth noting that a hypomorphic *IMP3* mutant allele in budding yeast sensitised cells to various DNA damaging agents and produced slightly longer telomeres [87]. Additional experiments will be needed to determine if this holds true in other organisms.

CCDC71 is an uncharacterized coiled-coil domain-containing protein for which there are no associated functions. Work done by the Human Protein Atlas program [55] suggests that the protein enriches at the nuclear periphery in HeLa and MCF7 cells and forms nuclear foci in U2OS cells. While it may be tempting to attribute the difference to the utilization of the ALT pathway, we found the antibody used in those experiments to also recognize several other proteins by western blotting. Our labeling of an epitope-tagged exogenous protein showed a pan-nuclear granular nuclear signal that was not exclusive of DAPI-dense regions (Fig 3E). Curated data suggest that CCDC71 binds histone H3.3 [52], components of the PRC2 complex [88], HDAC1 [89], and RBBP7 [89], linking the protein to chromatin compaction and transcriptional repression. We confirmed these interactions by immunoprecipitating the exogenous CCDC71 protein (S3(C) Fig). However, BioID provided important information on the most prominent associations (Fig 3F and S3(E) Fig). Our analysis clearly demonstrates a strong association between CCDC71 and HP1 isoforms, likely mediated by non-canonical PxVxL motifs in human cells (and canonical ones in mice) and/or through further associations with the related CCDC71L protein (Fig 3C and 3F, S3(A) and S3(E) Fig). In addition to confirming ATRX as a strong association, the CCDC71 BioID showed clear associations with the NAP1 histone chaperone and components of the NuRD chromatin-remodelling complex (which were also seen in the ATRX BioID, Fig 1B).

It is interesting to note that the ZFHX4 transcription factor (identified in the ATRX BioID) also interacts with NuRD to promote stem cell-like states [90]. Genetic deletions over the *ZFHX4* locus have been associated with syndromic Peters anomaly [91], ocular abnormalities [92], and intellectual disability [93], and the protein plays a role in maintaining the undifferentiated, self-renewing state of glioblastoma tumour-initiating cells [90]. Further studies will be needed to examine the effect of the ATRX-CCDC71-HP1, NAP1, and NuRD associations on chromatin. It will also be interesting to determine how these proteins (including those, such as the PRC2 complex, confirmed by immunoprecipitation in S3(C) Fig) functionally work together.

Chromatin maintenance is intimately tied to genome stability; hence, our investigations into the third chosen ATRX proximal association, SLF2. SLF2 associates with SLF1 and RAD18 and loads the cohesin-like SMC5/6 complex to promote genomic stability [44]. SLF1/2 knockdown impairs the recruitment of SMC5 to sites of DNA damage and increased the rate of global sister chromatid exchanges [44]. In contrast, SMC5/6 was found to promote the formation of APBs in ALT cells where it facilitates recombination between telomeres [49]. A tightly regulated process is therefore needed to prevent spurious recombination at telomeres. We saw evidence of ATRX-mediated recruitment of SLF2 to telomeres (Fig 4B), and found that the loss of the proteins causes telomere exchanges in HEK293 Flp-In cells (Fig 4D). SLF2 depletion was sufficient to induce high levels of telomere exchanges in U2OS cells (S4(E) Fig), showing that SLF1/2 is an important suppressor of recombination at telomeres.

Chromatin organization and maintenance by ATRX is needed to prevent gradual changes that enable the ALT phenotype [20]. To better understand how ATRX and SLF2 influence telomeres, we used RAP1 BioID to obtain information on changes caused by their loss. While few changes were observed upon loss of SLF2, intriguing changes were seen at telomeres approximately 15 passages after *ATRX* gene disruption (Figs 5C and 6). The changes seen in the single gene deletions were largely recapitulated in the double-null cells. Changes in chromatin remodelling, DNA replication, and repair were particularly notable. This opens the door to further explorations, especially those pertaining to ALT. For example, while a loss of ATRX does not in itself cause ALT, we saw increased levels of H2AX, PML, and the POLD3 subunit of DNA Pol*δ*, as well as lower levels of BRCA2 in ATRX-null cells (Fig 5C); trends seen in ALT cell models [13, 94, 95]. However, some of the strongest proteomic changes on telomeres caused by the loss of ATRX included a gain of a few zinc finger proteins and in the shugoshin 2 (SGO2) protein. While shugoshin proteins have centromeric functions, yeast Sgo2 is also needed for subtelomeric stability [96]. Contrastingly, the loss of ATRX also caused a notable depletion of core replisome proteins, such as MCM2–7 proteins and MCM10, from telomeres. Further deletion of SLF2 caused a number of changes that seemed additive (Fig 6), and this was sufficient to increase the number of telomere exchanges (Fig 4D). It is therefore likely that the changes on telomeric chromatin caused by the loss of ATRX potentiate the ALT phenotype through direct (e.g., loss of protein interactions) and indirect (e.g., downstream) effects.

**Fig 7:**
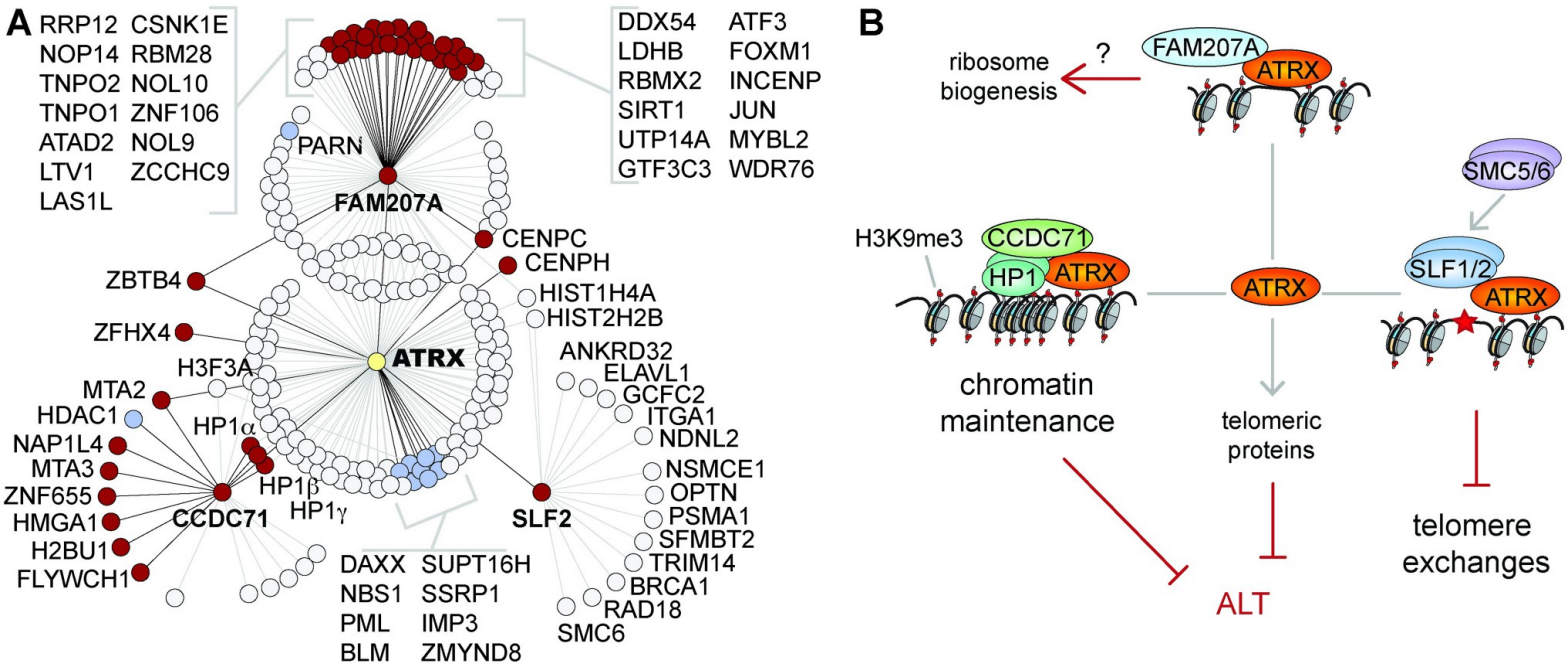
ATRX proteome overview and proposed model. A: Protein associations with ATRX, FAM207A, and CCDC71. Pale grey circles denote previously reported associations, while dark red circles represent newly identified proximal associations. Blue circles represent previously reported associations also identified by our BioID. Image was built using ProHits Viz [104] and the BioGrid database [52], and manually organized. B: Model proposing new biological roles for ATRX (other than histone deposition), mediated through proximal associations with FAM207A, CCDC71, and SLF2.

Clearly ATRX mediates multiple functions well beyond histone deposition with DAXX. Our data show that ATRX associates with a wide range of chromatin regulators with very different roles, as reflected by new and uncharacterized ATRX-associating proteins (Fig 7A). We propose that ATRX-associating proteins confer additional biological roles to protect repetitive DNA regions, and further impact chromatin in a manner that transcends ATRX’s immediate protein interactions (Fig 7B).

## Materials and methods

### Antibodies

The following primary antibodies were used: ATRX (Santa Cruz, sc-55584, PLA: 3 μg/mL, WB: 1:1000), ATRX (Santa Cruz, sc-15408, WB: 1:1000), ATRX (Thermo Fisher Scientific, PA5–21348, PLA: 1:1000–1:5000, WB: 1:1000), biotin (Cell Signaling Technology, 5597S, WB: 1:500), CCDC71 (Thermo Fisher Scientific, PA5–61543, PLA: 1:250), DAXX (Santa Cruz, sc-7152, PLA: 1:250, WB: 1:500), EZH2 (Sigma-Aldrich, MABE362, WB: 1:1000), FLAG (Sigma-Aldrich, F1804, IP: 2 μg/mg lysate, PLA: 1:1000, WB: 1:1000), GAPDH (Santa Cruz, sc-25778, WB: 1:1000), H3.3 (Abcam, ab176840, WB: 1:5000), HDAC1 (Thermo Fisher Scientific, PA1–860, WB: 1:1000), HP1 (Cell Signaling Technology, 2616S, WB: 1:1000), IMP3 (Thermo Fisher Scientific, PA5–26897, IF: 1:500, PLA: 1:500, WB: 1:1000), LaminB1 (Proteintech, 66095–1-Ig, WB: 1:50000), c-myc (Invitrogen, MA1–980, PLA: 1:2000), NBS1 (Novus Biologicals, NB100–143, PLA: 1:200, WB: 1:1000), SLF1 (Sigma-Aldrich, SAB2701555, WB: 1:1000), SLF2 (Thermo Fisher Scientific, PA5–66091, PLA: 2 μg/mL), SMC5 (Bethyl Laboratories, A300–236A, IF: 1:1000, WB: 1:1000), beta-tubulin (Developmental Studies Hybridoma Bank, E7, WB: 1:10000), ZBTB4 (Novus Biologicals, NBP1–76517, PLA: 1:10000), and ZFHX4 (Sigma-Aldrich, HPA023837, PLA: 1:500, WB: 1:500). The following secondary antibodies were used: anti-rabbit IgG HRP (Invitrogen, 0031458, WB: 1:10,000), anti-mouse kappa light chain HRP (Abcam, AB99617, WB: 1:10,000), anti-Rabbit IgG Alexa Fluor 594 (Jackson ImmunoResearch, 111–585-008, IF: 1:250), anti-rabbit IgG ATTO 647N (Rockland, 611–156-122S, IF: 1:250), anti-mouse IgG ATTO 488 (Rockland, 610–152-121S, IF: 1:250), and anti-mouse IgG AlexaFluor 647 (BioLegend, 405322, IF: 1:500).

### cDNA constructs

*ATRX* cDNA was amplified by PCR and the DNA band was excised and purified using the PureLink Gel Extraction Kit (Thermo Fisher Scientific, K210012) and introduced into pDONR223 using Gateway BP Clonase II Enzyme mix (Thermo Fisher Scientific, 8602289719), as well as a pFastBac vector (ThermoFisher Scientific) for expression in insect cells. pDONR223 plasmids containing CCDC71 and FAM207A were generated in the laboratory of Dr. Anne-Claude Gingras. Plasmids were prepared using Stable Competent E. coli (NEB, C3040I). Clones were selected from LB agar plates (1% tryptone, 0.5% yeast extract, 170 mM NaCl, pH 7.5) containing 50 μg/mL spectinomycin. *ATRX* was subcloned into a pDEST vector containing an N- or C-terminal FLAG-BirA* using Gateway LR Clonase II Enzyme mix (Thermo Fisher Scientific, 11791020). Transformed cells were selected on LB agar plates with 50 μg/mL ampicillin. The pDEST-pcDNA5-RAP1-BirA*-FLAG-C-term plasmid was prepared in the laboratory of Dr. Anne-Claude Gingras. Lentiviral particles encoding myc-tagged SLF2 were prepared in the laboratory of Dr. Grant Stewart.

### Cell lines and cell culture

HEK293 Flp-In T-REx cells (Thermo Fisher Scientific) were cultured in DMEM (Corning, 10–017-CV) supplemented with 10% FBS (Wisent, 080–150), 1X penicillin/streptomycin, 5 μg/mL blasticidin, and 75 μg/mL zeocin. HeLa S3 cells were grown in Joklik MEM (Caisson Labs), and MO3.13 (Cellutions Biosystems) in DMEM. Both were supplemented with bovine serum and penicillin/streptomycin. All cell lines were regularly tested for mycoplasma. Transfections were performed with lipofectamine 3000 (Thermo Fisher Scientific, L3000008) according to manufacturer’s instructions. For HEK293 Flp-In T-REx cells, transfections were performed at a 9:1 ratio of pOG44 to pDEST plasmid. Transfected HEK293 Flp-In cells (BirA*, NLS-BirA*, ATRX-BirA*, and RAP1-BirA*) were cultured in DMEM supplemented with 10% FBS, 1X penicillin/streptomycin, 5 μg/mL blasticidin, and 200 μg/mL hygromycin (for selection, or 100 μg/mL for maintenance). To generate ATRX KO lines, an sgRNA targeting exon 9 (5’- AAATTCCGAGTTTCGAGCGA -3’) was cloned into pSpCas9(BB)-2A-Puro (pX459) [97], a gift from Dr. Feng Zhang, Addgene plasmid 48139. For the SLF2 KO lines, sgRNA guides targeting exon 5 (5’- AGTTTCATCACTCGGTTCCT, GGCTTGGCACCTTCAAATTC -3’) were cloned into pSpCas9n(BB)-2A-GFP (pX461). Double ATRX/SLF2 KO cells were obtained by disrupting the *SLF2* gene in the *ATRX*-null cells. Constructs were transfected into HEK293 Flp-In RAP1-BirA* cells as described above. To validate the gene disruptions, genomic DNA was isolated by suspending cell pellets in genomic DNA extraction buffer (20 mM Tris pH 7.6, 150 mM KCl, 1 mM EDTA, 20% glycerol, 0.5% NP-40) supplemented with 1 mM DTT, 25 μg/mL RNase A, and 20 U/mL proteinase K and incubated at 60°C for 2 hr. DNA was extracted with phenol:chloroform and the disrupted genes were PCR amplified. PCR products were purified using PureLink PCR Purification Kit (Invitrogen, K310002) following the manufacturer’s protocol and prepared for Sanger sequencing using the BigDye Terminator v3.1 Cycle Sequencing Kit (Thermo Fisher, 4337457). Samples were sequenced on the Applied Biosystems SeqStudio Genetic Analyzer (Thermo Fisher). ATRX-null cells were further verified by western blotting. For SLF2, validation at the RNA level was done using PowerUp SYBR Green Master Mix (Applied Biosystems, A25742) according to manufacturer’s instructions, with the following primers: SLF2-RT-F 5’-AAACACTTTGTGCTACTCTGTGG-3’; SLF2-RT-R 5’-GTATCCTGGCGACCAAGTCTTTCA-3’; GAPDH-F 5’-CAATGACCCCTTCATTGACCTC-3’; and GAPDH-R 5’-GATCTCGCTCCTGGAAGATG-3’. Samples were analyzed with the QuantStudio 3 Real-Time PCR System (Applied Biosystems, A28567). Pyridostatin was purchased from Cayman Chemical (18013).

### BioID

Protocol was adapted from previously published work [98]. All BioID runs were performed in biological duplicates as previously described [98], but with results compared to 6 replicates of untransfected HEK293 Flp-In T-REx, 14 replicates of cells expressing BirA* and, where indicated, also 14 replicates of cells expressing BirA*-NLS. Less than 20 cell passages elapsed between the CRISPR-Cas9 gene disruptions and the RAP1 BioID experiments. Cells were seeded at 70% confluency and induced with 1 μg/mL tetracycline (or doxycycline concentrations yielding near equal protein expression across samples) for 25 hr and 50 μM biotin added during the last 8 hr. Cells were pelleted with at least 0.1 g per sample, and snap frozen. Upon processing, cells were lysed in modified RIPA buffer (50 mM Tris pH 7.5, 150 mM NaCl, 1.5 mM MgCl_2_, 1 mM EGTA, 0.1% SDS, 1% IGEPAL CA-630) with freshly added sodium deoxycholate (0.4%) and protease inhibitors (Sigma-Aldrich P8340) at 400 μL/0.1 g cells and solubilized for 20 min, gently rotating at 4°C (all the following 4°C incubations were also done with gentle rotation). Samples were sonicated at 25% amplitude for 5 sec on, 3 sec off cycles, for 3 cycles, using a Qsonica sonicator with CL-18 probe. Benzonase (Millipore 70746) was added and incubated for 15 min, 4°C (1.5 μL or 375 units per sample). Samples were spiked with additional SDS to a final concentration of 0.4% and incubated 15 min, 4°C. Samples were spun 16,000 x *g* for 20 min and the cleared lysates (supernatant) was transferred to a new tube. Streptavidin sepharose beads (GE 17–5113-01) were washed 3X with modified RIPA buffer with 0.4% SDS, and 30 μl (bed volume) was added to each sample and incubated 3 hr, 4°C. Samples were washed once with wash buffer (2% SDS, 50 mM Tris pH 7.5), 2X with modified RIPA buffer with 0.4% SDS, and 3X with ABC buffer (50 mM ammonium bicarbonate pH 8.5). Samples were spun, supernatant removed, and on-bead trypsin digest of peptides was performed by incubating with 1 μg trypsin dissolved in ABC buffer, rotating overnight at 37°C. An additional 0.5 μg trypsin per sample was added the next day and samples incubated for 2 hr at 37°C. Samples were gently vortexed, spun down, and supernatant transferred to new tube, with additional washing of beads and collection of supernatant performed twice (30 μL of HPLC grade water). Fresh 50% formic acid was added to samples to a final concentration of 2% prior to drying by vacuum centrifugation and subsequent storage at -80°C.

### Mass spectrometry acquisition

Each sample (6 μL in 2% formic acid; corresponding to 1/6th of a 15 cm tissue culture dish) was directly loaded at 800 nL/min onto an equilibrated HPLC column (pulled and packed in-house). The peptides were eluted from the column over a 90 min gradient generated by a Eksigent ekspert nanoLC 425 (Eksigent, Dublin CA) nano-pump and analysed on a TripleTOF 6600 instrument (AB SCIEX, Concord, Ontario, Canada). The gradient was delivered at 400 nL/min starting from 2% acetonitrile with 0.1% formic acid to 35% acetonitrile with 0.1% formic acid over 90 min followed by a 15 min clean-up at 80% acetonitrile with 0.1% formic acid, and a 15 min equilibration period back to 2% acetonitrile with 0.1% formic acid, for a total of 120 min. To minimize carryover between each sample, the analytical column was washed for 2 hr by running an alternating sawtooth gradient from 35% acetonitrile with 0.1% formic acid to 80% acetonitrile with 0.1% formic acid at a flow rate of 1500 nL/min, holding each gradient concentration for 5 min. Analytical column and instrument performance was verified after each sample by loading 30 fmol bovine serum albumin (BSA) tryptic peptide standard with 60 fmol alpha-casein tryptic digest and running a short 30 min gradient. TOF MS mass calibration was performed on BSA reference ions before running the next sample to adjust for mass drift and verify peak intensity. Samples were analyzed with two separate injections with instrument method set to data dependent acquisition (DDA) mode. The DDA method consisted of one 250 milliseconds (ms) MS1 TOF survey scan from 400–1800 Da followed by ten 100 ms MS2 candidate ion scans from 100–1800 Da in high sensitivity mode. Only ions with a charge of 2+ to 5+ that exceeded a threshold of 300 cps were selected for MS2, and former precursors were excluded for 7 seconds after one occurrence.

### Data-dependent acquisition data search

Mass spectrometry data generated were stored, searched, and analyzed using ProHits laboratory information management system (LIMS) platform [99]. Within ProHits, WIFF files were converted to an MGF format using the WIFF2MGF converter and to an mzML format using ProteoWizard (V3.0.10702) and the AB SCIEX MS Data Converter (V1.3 beta). The data were then searched using Mascot (V2.3.02) [100] and Comet (V2016.01 rev.2) [101]. The spectra were searched with the human and adenovirus sequences in the RefSeq database (version 57, January 30th, 2013) acquired from NCBI, supplemented with “common contaminants” from the Max Planck Institute (http://maxquant.org/contaminants.zip) and the Global Proteome Machine (GPM; ftp://ftp.thegpm.org/fasta/cRAP/crap.fasta), forward and reverse sequences (labeled “gi|9999” or “DECOY”), sequence tags (BirA, GST26, mCherry and GFP) and streptavidin, for a total of 72,481 entries. Database parameters were set to search for tryptic cleavages, allowing up to 2 missed cleavages sites per peptide with a mass tolerance of 35 ppm for precursors with charges of 2+ to 4+ and a tolerance of 0.15 amu for fragment ions. Variable modifications were selected for deamidated asparagine and glutamine and oxidized methionine. Results from each search engine were analyzed through TPP (the Trans-Proteomic Pipeline, v.4.7 POLAR VORTEX rev 1) via the iProphet pipeline [102].

### SAINT analysis

SAINTexpress version 3.6.1 [103] was used as a statistical tool to calculate the probability of potential protein-protein associations compared to background contaminants using default parameters, with bait compression set to 2 and control compression set to 4. A 95% FDR iProphet filter was used. SAINT scores with a Bayesian false discovery rate (BFDR) ≤ 1% were considered high-confidence protein interactions. All non-human protein interactors (did not start with “NP” in Prey column) were removed from the SAINT analysis, except for BirA R118G H0QFJ5. Dot plots were generated using the “Dot plot generator” tool in ProHits-viz [104] using SAINTexpress file generated from ProHits. Data were normalized by total abundance. Gene ontology term analysis for biological process (GO:BP) was done using g:Profiler [105].

### Proximity ligation assay

Cells were seeded to a density of 50–70% on 12 mm glass coverslips coated in poly-L-lysine (Sigma-Aldrich, P8920). Cells were fixed with a 2% PFA solution (2% PFA, 0.2% Triton-X-100, pH 8.2), washed with 1X PBS (137 mM NaCl, 2.7 mM KCl, 10 mM Na_2_HPO_4_, 1.8 mM KH_2_PO_4_), permeabilized with 0.5% tergitol (Sigma-Aldrich, NP40S), and washed with 1X PBS. PLAs were performed according to manufacturer’s instructions using the Duolink *In Situ* Detection Kit (Sigma-Aldrich, DUO92013). Confocal microscopy was done at the SickKids Imaging Facility and foci counted using CellProfiler [106]. Signal intensity was obtained using ImageJ, with background signal subtracted for each figure [107].

### Cell lysis & subcellular fractionation

All steps below were performed at 4°C. Subcellular fractionation was done by gently dounce homogenizing PBS-washed cell pellets in 2 pellet volumes of cytosolic lysis buffer (20 mM Tris pH 7.6, 10 mM NaCl, 1 mM EDTA, 0.5% IGEPAL, 0.5 mM DTT, protease inhibitors) using a loose pestle. Nuclei were recovered by centrifugation at 500 x *g* for 5 min, and the supernatant containing the cytoplasmic fraction was transferred to a new tube. Nuclei were then resuspended in 3 pellet volumes of nuclear extraction buffer (20 mM Tris pH 7.6, 300 mM NaCl, 1 mM EDTA, 20% glycerol, 0.5 mM DTT) with protease inhibitors and dounce homogenized with a tight pestle. The mixture was spun down at 30,000 x *g* for 15 min, and the supernatant containing nuclear extracts was transferred to a fresh tube. The remaining insoluble pellet was resuspended in 3 volumes of BC100 (20 mM Tris pH 7.6, 100 mM NaCl, 0.2 mM EDTA, 20% glycerol, 0.5 mM DTT) with protease inhibitors. The sample was then sonicated and digested with 75 U/μL MNase in the presence of 5 mM CaCl_2_ for 2 hr, with gentle rotation. The subcellular fractions were dialyzed against BC100 and insoluble material removed by centrifugation as per conditions above. Whole cell extracts were generated by incubating PBS-washed cell pellets with 3 pellet volumes of lysis buffer (50 mM Tris pH 7.6, 100 mM KCl, 2 mM EDTA, 0.1% NP-40, 10% glycerol, 0.5 mM DTT). Samples were then sonicated, digested and processed as per the solubilized nuclear fraction above.

### Immunoprecipitation

Cells were seeded at 4 x 10^6^ cells per plate and induced with 1 μg/mL doxycycline for 24 hr. The cells were harvested by first washing with cold 1X PBS, scraping, and collecting the cells in 1 mL 1X PBS. The cells were spun down at 500 x *g* for 5 min at 4ºC, and lysed in 1X IP lysis buffer (50 mM Tris pH 7.6, 100 mM KCl, 2 mM EDTA, 0.1% IGEPAL, 10% glycerol). The lysate was flash frozen on dry ice for 10 min and then allowed to thaw at room temperature. The lysate was then sonicated 3X for 10 sec at 1.5 amp. After sonication, the lysate was digested with 1 U/μL MNase and the buffer supplemented with 5 mM CalCl_2_. This was left to incubate overnight with rocking at 4ºC. Four milligrams of protein was incubated with anti-FLAG (1 μg/mg lysate) overnight at 4ºC, rocking. The following day, magnetic Protein G beads (Cytiva, 28967070) were washed three times in 1X IP lysis buffer and the lysate and antibody mix was added onto the beads and incubated at 4ºC for 4 hr with rocking. The supernatant was removed, and the beads were washed three times in 1X IP lysis buffer. Proteins were eluted with 40 μL 1X Laemmli (2% SDS, 0.1% bromophenol blue, 10% glycerol, 62.5 mM Tris pH 6.8, 100 mM DTT) and boiled at 95ºC for 15 min.

### *In vitro* transcription/translation & pulldown assays

FLAG-ATRX was expressed from baculovirus infected SF9 insect cells as per previous descriptions [108]. The protein was immobilized on an M2 resin (MilliporeSigma), washed, and eluted with 25 μg/ml 3X FLAG peptide (MilliporeSigma). The TnT Quick Coupled Transcription/Translation System (Promega, L1170) was used to generate FAM207A in accordance with manufacturer’s instructions, with the addition of Transcend tRNA (Promega L506A) and 1 μg/mL leupeptin. Recombinant FAM207A was incubated with Strep-Tactin MacroPrep resin (IBA LifeSciences 2–1505-010) in BC150 (20 mM Tris pH 7.6, 150 mM NaCl, 0.2 mM EDTA, 20% glycerol) for 30 min at 4°C prior to the addition of 20 pmol of recombinant ATRX. Proteins were incubated together at room temperature for 1 hr before washing with BC150 + 0.1% NP40, BC300 (20 mM Tris pH 7.6, 300 mM NaCl, 0.2 mM EDTA, 20% glycerol) + 0.1% NP40, and BC150. Samples were spiked with 1X Laemmli buffer and boiled at 95°C for 10 min prior to Western blotting.

### Western blotting

Protein lysates were diluted in 1X Laemmli buffer and boiled at 95°C for 5 min. For streptavidin pull downs, 2 mM biotin was added to 1X Laemmli prior to boiling. Protein lysates were loaded onto 8–12% Tris-Glycine gels and run at 100 V for 90 min in 1X running buffer (25 mM Tris, 192 mM glycine, 0.1% SDS). Proteins were then transferred onto activated PVDF membranes for 2 hr at 270 mA in 1X transfer buffer (200 mM glycine, 25 mM Tris) using a Mini-protean wet transfer system (BioRad). All samples were run this way, with the exception of ZFHX4 fractionations, which were loaded onto a 3–8% Tris-Acetate gel (Thermo Fisher, EA0375PK2), run at 125V for 1.5 hr in 1X running buffer (Invitrogen, LA0041) supplemented with 500 μL antioxidant (Thermo Fisher, NP0005). Gels were washed in 20% ethanol for 10 min prior to transfer to PVDF with the iBlot (Thermo, IB21001) at 20V for 2 min, 23V for 6 min, and 25V for 4 min. All membranes were blocked in 5% milk in 1X TBST (20 mM Tris, 150 mM NaCl, 0.1% Tween) and incubated with primary antibody diluted in 1X TBST and 0.04% NaN_3_ for 1 hr at room temperature, or overnight at 4°C with gentle rotation. Membranes were then washed 3X for 5 min with 1X TBST and incubated with HRP-conjugated secondary antibodies diluted in 1X TBST with 5% milk at room temperature, rocking for 1 hr. Membranes were then washed 3X 5 min with 1X TBST. To image, a 5X luminol solution (100 mM Tris pH 8.8, 1.25 mM luminol, 0.2 mM coumaric acid) was diluted as needed and supplemented with 5 μL 10% H_2_O_2_ per mL, and poured over the blot. For westerns of the TnT reactions, samples were treated as described above, with the exception of the luminol solution. Instead, blots were washed treated with streptavidin-AP (Promega V5591) according to manufacturer’s instructions, rocking at room temperature for 1 hr. Blots were washed twice with 1X TBST and then twice with water. Blots were then incubated in Western Blue Stabilized Substrate (Promega S384C) according to manufacturer’s instructions. Images were captured using a BioRad ChemiDoc XRS+ system.

### CO-FISH

CO-FISH was performed as previously described [109] with the following modifications. Cells were treated with 10 μM BrdU for 18 hr and 0.2 μg/mL KaryoMAX Colcemid (Thermo Fisher, 15212012) for the final 4 hr. Metaphase spreads were prepared using a CDS 5 Cytogenetic Drying chamber (Thermotron) according to standard methods, rehydrated with 1X PBS, and slides were treated with 0.5 mg/mL RNase A (Life Technologies, 12091021) at 37°C for 10 min. Slides were stained with 0.5 μg/mL bisbenzimide Hoechst 33258 (Sigma-Aldrich, 14530) in 2X SSC (300 mM NaCl, 30 mM sodium citrate) for 15 min at room temperature. Slides were exposed to 365 nm UV, 5.4 x 10^3^ J/m2 twice. Next, slides were treated with 10 U/μL exonuclease III (NEB, M0206L) for 40 min at 37°C. Cells were washed in 1X PBS and then dehydrated in a series of 70, 95, and 100% ethanol. TelG-Cy3 (PNA Bio, F1006) and TelC-ALEXA 488 (PNA Bio, F1004) probes were heated at 70°C for 30 min, diluted in hybridization buffer [20 mM Tris pH 7.2, 60% formamide (Thermo Fisher, 15515026), 0.5% block (Roche, 11096176001)], and applied to slides for 2 hr each at room temperature, sequentially. Slides were washed with Wash Buffer 1 (10 mM Tris pH 7.2, 70% formamide, 0,1% BSA) and Wash Buffer 2 (100 mM Tris pH 7.2, 150 mM NaCl, 0.08% Tween-20) three times each. DAPI (Sigma-Aldrich, D9542) was added at final concentration of 1 μg/mL to the second wash with Wash Buffer 2. Then, slides were dehydrated in an ethanol series, as before, and mounted with ProLong Gold Antifade Mountant (Invitrogen, P36930). Slides were imaged with an Olympus BX61 microscope with the CytoPower automated imaging system (Applied Spectral Imaging). Telomere exchanges were manually scored in a blinded fashion, with sample identity only revealed after all samples were scored.

### Immunofluorescence

Cells were seeded on coverslips and fixed in a 2% PFA solution for 20 min at room temperature after the desired treatment. Cells were washed 3X in 1X PBS and permeabilised with 0.5% tergitol in 1X PBS for 10 min at room temperature. Coverslips were washed 3X in 1X PBS and incubated in blocking buffer (3% BSA, 1% NGS, 1x PBS) for 1 hr at room temperature. Cells were then incubated in primary antibody diluted in blocking buffer for 1.5 hr at room temperature in a humidity chamber, washed 3X in 1X PBS, and incubated in secondary antibody diluted in blocking buffer for 30 min at room temperature. Coverslips were washed 3X with 1X PBS and treated with 1 μg/mL DAPI diluted in 1X PBS for 10 min at room temperature. Coverslips were washed once more in 1X PBS, mounted onto slides and imaged as described for the PLAs.

### IF-FISH

Cells were seeded on slides, fixed in a 2% PFA solution for 10 min at room temperature, washed 3X in 1X PBS, and treated with 0.5% tergitol in 1X PBS for 10 min at room temperature. Cells were washed 3X in 1X PBS and incubated in blocking buffer for 1 hr at room temperature. Cells were then incubated in primary antibody diluted in blocking buffer for 1.5 hr at room temperature in a humidity chamber, washed 3X in 1X PBS, and incubated in secondary antibody diluted in blocking buffer for 30 min at room temperature. Cells were washed 2X with 1X PBS and fixed in 2% PFA in 1X PBS for 10 min at room temperature. Slides were dehydrated in an ethanol series of 70, 95, and 100% for 5 min each. Slides were air dried and TelC-488 probe (PNA Bio, F1004) was heated at 72°C for 30 min before being diluted 1:500 in hybridization buffer [70% formamide, 0.5% blocking reagent (Roche, 11096176001), 10 mM Tris pH 7.2] and being applied to slides. Slides were heated at 72°C for 10 min, sealed with rubber cement, and placed in a humidity chamber to allow probe binding overnight. The next day, slides were washed 2X, 15 min each at room temperature in wash buffer 1 (70% formamide, 10 mM Tris pH 7.2). Slides were then washed 3X, 5 min each at room temperature in wash buffer 2 (100 mM Tris pH 7.2, 150 mM NaCl, 0.08% Tween 20), with 1 μg/mL of DAPI being added to the second wash. Slides were dehydrated in an ethanol series as before, allowed to air dry, and mounted before being imaged. Colocalization was scored using the Cellprofiler Colocalization pipeline [106] scoring foci that were 2–15 pixels in diameter and greater than 0.15 units of intensity.

## Supporting information

S1 Fig ATRX BioID system and proximal associations. A: Western blot exemplifying stable integration and expression of FLAG-tagged BirA* protein fusions in inducible HEK293 Flp-In T-REx cells (top), and similar expression levels of N- and C-terminally-tagged ATRX (bottom). B: Western blot (top) and Coomassie-staining (bottom) of biotinylated proteins captured on streptavidin beads. C: Example of immunofluorescent labeling demonstrating nuclear targeting of the ATRX-BirA* fusion constructs. D: Gene ontology analysis of ATRX-associating proteins. E: Western blot showing the subcellular distribution of ATRX-associating proteins in HeLa S3 cells. F: Examples of the proximity ligation assay (PLA) showing associations between endogenous proteins in MO3.13 cells. G-H: PLAs showing associations between endogenous ATRX and exogenous FLAG-tagged CCDC71 (G) or FAM207A (H) in HEK293 Flp-In T-REx cells. I: PLAs showing an association between endogenous ATRX and myc-tagged SLF2. Data plots account for ∼100 nuclei in three independent experiments, with the total number of nuclei assessed in brackets. The p-values were obtained using a 2-sided Student’s t-test with unequal variance. J: *In vitro* interaction between recombinant FAM207A and ATRX.
(PDF)

S2 Fig FAM207A subcellular expression and BioID system. A: Subcellular fractionation of HEK293 Flp-In T-REx cells expressing FLAG-FAM207A. Cy—cytoplasm; Nu—nucleus; Ch—chromatin. B: Western analysis of BirA* constructs used for the FAM207A BioID experiment. Duplicate (induced) lanes are shown. C: Immunolabeling experiment showing co-localization of FAM207A and the IMP3 nucleolar protein. Scale bar = 4μm. D: Dot plot showing prey proteins identified with FAM207A-BirA* that were enriched over endogenous biotinylation (untransfected), unspecific pan-cellular biotinylation (BirA*), and unspecific nuclear biotinylation (NLS-BirA*; BFDR ≤ 5%, SAINT [103]). Data represent two biological replicates. Proteins in boldface remained statistically enriched when the nuclear localization signal (NLS)-BirA* control was used to further filter the FAM207A BioID data. E: Full western blots for Fig 2A. The red boxes indicate the areas shown in the main figure.
(PDF)

S3 Fig PxVxL motif in CCDC71L, CCDC71 subcellular fractionation and immunoprecipitation, and BioID system. A: PxVxL motif in human CCDC71L. B: Subcellular fractionation of HEK293 Flp-In T-REx cells expressing FLAG-CCDC71. Cy—cytoplasm; Nu—nucleus; Ch—chromatin. C: CCDC71 immunoprecipitation and verification of associations reported in BioGrid [52]. D: Western analysis of CCDC71 and control BirA* constructs. E: Dot plot showing prey proteins identified with CCDC71-BirA* that were enriched over endogenous biotinylation (untransfected) and unspecific pan-cellular biotinylation (BirA*; BFDR ≤ 5%, SAINT [103]). Data represent two biological replicates. Proteins in boldface remained statistically enriched when the nuclear localization signal (NLS)-BirA* control was used to further filter the CCDC71 BioID data. F: Full western blots for Fig 3D. The red boxes indicate the areas shown in the main figure.
(PDF)

S4 Fig Co-localization of SLF2 and telomeres, SLF2-null HEK293 Flp-In T-REx cells, and telomere exchanges in U2OS cells. A: Western blot showing exogenous myc-SLF2 expression in lentivirus-infected HEK293 Flp-In T-REx (with inducible RAP1-BirA*, but the latter is not induced). B: Immunolabeling of myc-SLF2 (yellow) and SMC5 (red), and fluorescence *in situ* hybridization (IF-FISH) using a telomeric (green) probe in ATRX-expressing and -null HEK293 Flp-In cells. C: CRISPR-Cas9-mediated *SLF2* gene disruptions in HEK293 Flp-In T-REx (RAP1-BirA*), detected by DNA sequencing, and matching qRT-PCR (D). E: Telomere exchange rates observed in U2OS cells. At least 30 mitotic spreads per condition were counted and the percent of telomeric exchanges plotted. F: Full western blots for Fig 4A. The red boxes indicate the areas shown in the main figure.
(PDF)

S5 Fig RAP1 BioID and SLF2-null cells. A: Western blot showing the induction of FLAG- and BirA*-tagged RAP1 in ATRX-expressing and -null HEK293 Flp-In T-REx cells. B: Western analysis of shRNA-mediated ATRX knockdown in HEK293 Flp-In T-REx cells expressing RAP1-BirA*. Doubly transfected cells (shRNA constructs 1 and 2) were used for the RAP1 BioID. C: Overlap between RAP1 BioID and TERF1 BioID [65], dCas9-APEX2 biotinylation at genomic elements by restricted spatial tagging (C-BERST) [68], quantitative telomeric chromatin isolation protocol (QTIP) [67], and proteomics of isolated chromatin segments (PICh) [66]. PICh and QTIP data were grouped to simplify the Venn diagrams. Data consider HEK293 Flp-In cells expressing ATRX (unperturbed). A list of proteins commonly identified in at least three different telomere proteomic screens is shown on the right. D: Fold change of prey proteins identified by RAP1 BioID (BFDR ≤ 5%, SAINT [103]) in ATRX KD vs. ATRX-expressing HEK293 Flp-In cells. Labeled proteins had ≥2.5 average spectra and ≥1.5-fold change between conditions. Proteins labeled in the scatter plot remained significant when NLS-BirA* was used as a control. Inset box represents proteins also identified with NLS-BirA* as a bait (less stringent).
(PDF)

S1 Data BioID analysis. (XLSX)

S2 Data Data for plots other than BioID. (XLSX)

## References

[1] Wong LH, McGhie JD, Sim M, Anderson MA, Ahn S, Hannan RD, George AJ, Morgan KA, Mann JR, Choo KH. ATRX interacts with H3.3 in maintaining telomere structural integrity in pluripotent embryonic stem cells. Genome Res. 2010 Mar;20(3):351–60. doi: 10.1101/gr.101477.109 20110566 PMC2840985

[2] Emelyanov AV, Konev AY, Vershilova E, Fyodorov DV. Protein complex of Drosophila ATRX/XNP and HP1a is required for the formation of pericentric beta-heterochromatin in vivo. J Biol Chem. 2010 May 14;285(20):15027–15037. doi: 10.1074/jbc.M109.064790 20154359 PMC2865330

[3] Voon HP, Hughes JR, Rode C, De La Rosa-Velázquez IA, Jenuwein T, Feil R, Higgs DR, Gibbons RJ. ATRX Plays a Key Role in Maintaining Silencing at Interstitial Heterochromatic Loci and Imprinted Genes. Cell Rep. 2015 Apr 21;11(3):405–18. doi: 10.1016/j.celrep.2015.03.036 25865896 PMC4410944

[4] Juhász S, Elbakry A, Mathes A, Löbrich M. ATRX Promotes DNA Repair Synthesis and Sister Chromatid Exchange during Homologous Recombination. Mol Cell. 2018 Jul 5;71(1):11–24.e7. doi: 10.1016/j.molcel.2018.05.014 29937341

[5] Watson LA, Solomon LA, Li JR, Jiang Y, Edwards M, Shin-ya K, Beier F, Bérubé NG. Atrx deficiency induces telomere dysfunction, endocrine defects, and reduced life span. J Clin Invest. 2013 May;123(5):2049–63. doi: 10.1172/JCI65634 23563309 PMC3635723

[6] Gibbons RJ, Picketts DJ, Villard L, Higgs DR. Mutations in a putative global transcriptional regulator cause X-linked mental retardation with alpha-thalassemia (ATR-X syndrome). Cell. 1995 Mar 24;80(6):837–45. doi: 10.1016/0092-8674(95)90287-2 7697714

[7] Heaphy CM, de Wilde RF, Jiao Y, Klein AP, Edil BH, Shi C, Bettegowda C, Rodriguez FJ, Eberhart CG, Hebbar S, Offerhaus GJ, McLendon R, Rasheed BA, He Y, Yan H, Bigner DD, Oba-Shinjo SM, Marie SK, Riggins GJ, Kinzler KW, Vogelstein B, Hruban RH, Maitra A, Papadopoulos N, Meeker AK. Altered telomeres in tumors with ATRX and DAXX mutations. Science. 2011 Jul 22;333(6041):425. doi: 10.1126/science.1207313 21719641 PMC3174141

[8] Lovejoy CA, Li W, Reisenweber S, Thongthip S, Bruno J, de Lange T, De S, Petrini JH, Sung PA, Jasin M, Rosenbluh J, Zwang Y, Weir BA, Hatton C, Ivanova E, Macconaill L, Hanna M, Hahn WC, Lue NF, Reddel RR, Jiao Y, Kinzler K, Vogelstein B, Papadopoulos N, Meeker AK; ALT Starr Cancer Consortium. Loss of ATRX, genome instability, and an altered DNA damage response are hallmarks of the alternative lengthening of telomeres pathway. PLoS Genet. 2012;8(7):e1002772. doi: 10.1371/journal.pgen.1002772 22829774 PMC3400581

[9] Clynes D, Jelinska C, Xella B, Ayyub H, Scott C, Mitson M, Taylor S, Higgs DR, Gibbons RJ. Suppression of the alternative lengthening of telomere pathway by the chromatin remodelling factor ATRX. Nat Commun. 2015 Jul 6;6:7538. doi: 10.1038/ncomms8538 26143912 PMC4501375

[10] Heaphy CM, Subhawong AP, Hong SM, Goggins MG, Montgomery EA, Gabrielson E, Netto GJ, Epstein JI, Lotan TL, Westra WH, Shih IeM, Iacobuzio-Donahue CA, Maitra A, Li QK, Eberhart CG, Taube JM, Rakheja D, Kurman RJ, Wu TC, Roden RB, Argani P, De Marzo AM, Terracciano L, Torbenson M, Meeker AK. Prevalence of the alternative lengthening of telomeres telomere maintenance mechanism in human cancer subtypes. Am J Pathol. 2011 Oct;179(4):1608–15. doi: 10.1016/j.ajpath.2011.06.018 21888887 PMC3181356

[11] Bryan TM, Englezou A, Gupta J, Bacchetti S, Reddel RR. Telomere elongation in immortal human cells without detectable telomerase activity. EMBO J. 1995 Sep 1;14(17):4240–8. doi: 10.1002/j.1460-2075.1995.tb00098.x 7556065 PMC394507

[12] Episkopou H, Draskovic I, Van Beneden A, Tilman G, Mattiussi M, Gobin M, Arnoult N, Londoño-Vallejo A, Decottignies A. Alternative Lengthening of Telomeres is characterized by reduced compaction of telomeric chromatin. Nucleic Acids Res. 2014 Apr;42(7):4391–405. doi: 10.1093/nar/gku114 24500201 PMC3985679

[13] Yeager TR, Neumann AA, Englezou A, Huschtscha LI, Noble JR, Reddel RR. Telomerase-negative immortalized human cells contain a novel type of promyelocytic leukemia (PML) body. Cancer Res. 1999 Sep 1;59(17):4175–9. 10485449

[14] Londoño-Vallejo JA, Der-Sarkissian H, Cazes L, Bacchetti S, Reddel RR. Alternative lengthening of telomeres is characterized by high rates of telomeric exchange. Cancer Res. 2004 Apr 1;64(7):2324–7. doi: 10.1158/0008-5472.CAN-03-4035 15059879

[15] Lovejoy CA, Takai K, Huh MS, Picketts DJ, de Lange T. ATRX affects the repair of telomeric DSBs by promoting cohesion and a DAXX-dependent activity. PLoS Biol. 2020 Jan 2;18(1):e3000594. doi: 10.1371/journal.pbio.3000594 31895940 PMC6959610

[16] Sobinoff AP, Allen JA, Neumann AA, Yang SF, Walsh ME, Henson JD, Reddel RR, Pickett HA. BLM and SLX4 play opposing roles in recombination-dependent replication at human telomeres. EMBO J. 2017 Oct 2;36(19):2907–2919. doi: 10.15252/embj.201796889 28877996 PMC5623873

[17] Cesare AJ, Griffith JD. Telomeric DNA in ALT cells is characterized by free telomeric circles and heterogeneous t-loops. Mol Cell Biol. 2004 Nov;24(22):9948–57. doi: 10.1128/MCB.24.22.9948-9957.2004 15509797 PMC525488

[18] Sobinoff AP, Pickett HA. Mechanisms that drive telomere maintenance and recombination in human cancers. Curr Opin Genet Dev. 2020 Feb;60:25–30. doi: 10.1016/j.gde.2020.02.006 32119936

[19] Kent T, Gracias D, Shepherd S, Clynes D. Alternative Lengthening of Telomeres in Pediatric Cancer: Mechanisms to Therapies. Front Oncol. 2020 Jan 21;9:1518. doi: 10.3389/fonc.2019.01518 32039009 PMC6985284

[20] Li F, Deng Z, Zhang L, Wu C, Jin Y, Hwang I, Vladimirova O, Xu L, Yang L, Lu B, Dheekollu J, Li JY, Feng H, Hu J, Vakoc CR, Ying H, Paik J, Lieberman PM, Zheng H. ATRX loss induces telomere dysfunction and necessitates induction of alternative lengthening of telomeres during human cell immortalization. EMBO J. 2019 Oct 1;38(19):e96659. doi: 10.15252/embj.201796659 31454099 PMC6769380

[21] Napier CE, Huschtscha LI, Harvey A, Bower K, Noble JR, Hendrickson EA, Reddel RR. ATRX represses alternative lengthening of telomeres. Oncotarget. 2015 Jun 30;6(18):16543–58. doi: 10.18632/oncotarget.3846 26001292 PMC4599288

[22] Cesare AJ, Kaul Z, Cohen SB, Napier CE, Pickett HA, Neumann AA, Reddel RR. Spontaneous occurrence of telomeric DNA damage response in the absence of chromosome fusions. Nat Struct Mol Biol. 2009 Dec;16(12):1244–51. doi: 10.1038/nsmb.1725 19935685

[23] Zhang JM, Genois MM, Ouyang J, Lan L, Zou L. Alternative lengthening of telomeres is a self-perpetuating process in ALT-associated PML bodies. Mol Cell. 2021 Mar 4;81(5):1027–1042.e4. doi: 10.1016/j.molcel.2020.12.030 33453166 PMC8245000

[24] Hoang SM, O’Sullivan RJ. Alternative Lengthening of Telomeres: Building Bridges To Connect Chromosome Ends. Trends Cancer. 2020 Mar;6(3):247–260. doi: 10.1016/j.trecan.2019.12.009 32101727 PMC7199893

[25] Wu G, Jiang X, Lee WH, Chen PL. Assembly of functional ALT-associated promyelocytic leukemia bodies requires Nijmegen Breakage Syndrome 1. Cancer Res. 2003 May 15;63(10):2589–95. 12750284

[26] Jiang WQ, Zhong ZH, Henson JD, Neumann AA, Chang AC, Reddel RR. Suppression of alternative lengthening of telomeres by Sp100-mediated sequestration of the MRE11/RAD50/NBS1 complex. Mol Cell Biol. 2005 Apr;25(7):2708–21. doi: 10.1128/MCB.25.7.2708-2721.2005 15767676 PMC1061646

[27] Wu G, Lee WH, Chen PL. NBS1 and TRF1 colocalize at promyelocytic leukemia bodies during late S/G2 phases in immortalized telomerase-negative cells. Implication of NBS1 in alternative lengthening of telomeres. J Biol Chem. 2000 Sep 29;275(39):30618–22. doi: 10.1074/jbc.C000390200 10913111

[28] Syed A, Tainer JA. The MRE11-RAD50-NBS1 Complex Conducts the Orchestration of Damage Signaling and Outcomes to Stress in DNA Replication and Repair. Annu Rev Biochem. 2018 Jun 20;87:263–294. doi: 10.1146/annurev-biochem-062917-012415 29709199 PMC6076887

[29] Zhong ZH, Jiang WQ, Cesare AJ, Neumann AA, Wadhwa R, Reddel RR. Disruption of telomere maintenance by depletion of the MRE11/RAD50/NBS1 complex in cells that use alternative lengthening of telomeres. J Biol Chem. 2007 Oct 5;282(40):29314–22. doi: 10.1074/jbc.M701413200 17693401

[30] Leung JW, Ghosal G, Wang W, Shen X, Wang J, Li L, Chen J. Alpha thalassemia/mental retardation syndrome X-linked gene product ATRX is required for proper replication restart and cellular resistance to replication stress. J Biol Chem. 2013 Mar 1;288(9):6342–50. doi: 10.1074/jbc.M112.411603 23329831 PMC3585069

[31] Clynes D, Jelinska C, Xella B, Ayyub H, Taylor S, Mitson M, Bachrati CZ, Higgs DR, Gibbons RJ. ATRX dysfunction induces replication defects in primary mouse cells. PLoS One. 2014 Mar 20;9(3):e92915. doi: 10.1371/journal.pone.0092915 24651726 PMC3961441

[32] Iwase S, Xiang B, Ghosh S, Ren T, Lewis PW, Cochrane JC, Allis CD, Picketts DJ, Patel DJ, Li H, Shi Y. ATRX ADD domain links an atypical histone methylation recognition mechanism to human mental-retardation syndrome. Nat Struct Mol Biol. 2011 Jun 12;18(7):769–76. doi: 10.1038/nsmb.2062 21666679 PMC3130887

[33] Dhayalan A, Tamas R, Bock I, Tattermusch A, Dimitrova E, Kudithipudi S, Ragozin S, Jeltsch A. The ATRX-ADD domain binds to H3 tail peptides and reads the combined methylation state of K4 and K9. Hum Mol Genet. 2011 Jun 1;20(11):2195–203. doi: 10.1093/hmg/ddr107 21421568 PMC3090196

[34] Eustermann S, Yang JC, Law MJ, Amos R, Chapman LM, Jelinska C, Garrick D, Clynes D, Gibbons RJ, Rhodes D, Higgs DR, Neuhaus D. Combinatorial readout of histone H3 modifications specifies localization of ATRX to heterochromatin. Nat Struct Mol Biol. 2011 Jun 12;18(7):777–82. doi: 10.1038/nsmb.2070 21666677

[35] Lechner MS, Schultz DC, Negorev D, Maul GG, Rauscher FJ 3rd. The mammalian heterochromatin protein 1 binds diverse nuclear proteins through a common motif that targets the chromoshadow domain. Biochem Biophys Res Commun. 2005 Jun 17;331(4):929–37. doi: 10.1016/j.bbrc.2005.04.016 15882967

[36] Drané P, Ouararhni K, Depaux A, Shuaib M, Hamiche A. The death-associated protein DAXX is a novel histone chaperone involved in the replication-independent deposition of H3.3. Genes Dev. 2010 Jun 15;24(12):1253–65. doi: 10.1101/gad.566910 20504901 PMC2885661

[37] Lewis PW, Elsaesser SJ, Noh KM, Stadler SC, Allis CD. Daxx is an H3.3-specific histone chaperone and cooperates with ATRX in replication-independent chromatin assembly at telomeres. Proc Natl Acad Sci U S A. 2010 Aug 10;107(32):14075–80. doi: 10.1073/pnas.1008850107 20651253 PMC2922592

[38] Elsässer SJ, Noh KM, Diaz N, Allis CD, Banaszynski LA. Histone H3.3 is required for endogenous retroviral element silencing in embryonic stem cells. Nature. 2015 Jun 11;522(7555):240–244. doi: 10.1038/nature14345 25938714 PMC4509593

[39] Udugama M, Chang FTM, Chan FL, Tang MC, Pickett HA, McGhie JDR, Mayne L, Collas P, Mann JR, Wong LH. Histone variant H3.3 provides the heterochromatic H3 lysine 9 tri-methylation mark at telomeres. Nucleic Acids Res. 2015 Dec 2;43(21):10227–37. doi: 10.1093/nar/gkv847 26304540 PMC4666390

[40] Ramamoorthy M, Smith S. Loss of ATRX Suppresses Resolution of Telomere Cohesion to Control Recombination in ALT Cancer Cells. Cancer Cell. 2015 Sep 14;28(3):357–69. doi: 10.1016/j.ccell.2015.08.003 26373281 PMC4573400

[41] Hein MY, Hubner NC, Poser I, Cox J, Nagaraj N, Toyoda Y, Gak IA, Weisswange I, Mansfeld J, Buchholz F, Hyman AA, Mann M. A human interactome in three quantitative dimensions organized by stoichiometries and abundances. Cell. 2015 Oct 22;163(3):712–23. doi: 10.1016/j.cell.2015.09.053 26496610

[42] Roux KJ, Kim DI, Raida M, Burke B. A promiscuous biotin ligase fusion protein identifies proximal and interacting proteins in mammalian cells. J Cell Biol. 2012 Mar 19;196(6):801–10. doi: 10.1083/jcb.201112098 22412018 PMC3308701

[43] Scott WA, Campos EI. Interactions With Histone H3 and Tools to Study Them. Front Cell Dev Biol. 2020 Jul 31;8:701. doi: 10.3389/fcell.2020.00701 32850821 PMC7411163

[44] Räschle M, Smeenk G, Hansen RK, Temu T, Oka Y, Hein MY, Nagaraj N, Long DT, Walter JC, Hofmann K, Storchova Z, Cox J, Bekker-Jensen S, Mailand N, Mann M. DNA repair. Proteomics reveals dynamic assembly of repair complexes during bypass of DNA cross-links. Science. 2015 May 1;348(6234):1253671. doi: 10.1126/science.1253671 25931565 PMC5331883

[45] Torres-Rosell J, Machín F, Farmer S, Jarmuz A, Eydmann T, Dalgaard JZ, Aragón L. SMC5 and SMC6 genes are required for the segregation of repetitive chromosome regions. Nat Cell Biol. 2005 Apr;7(4):412–9. doi: 10.1038/ncb1239 15793567

[46] Aragón L. The Smc5/6 Complex: New and Old Functions of the Enigmatic Long-Distance Relative. Annu Rev Genet. 2018 Nov 23;52:89–107. doi: 10.1146/annurev-genet-120417-031353 30476445

[47] Gutierrez-Escribano P, Hormeño S, Madariaga-Marcos J, Solé-Soler R, O’Reilly FJ, Morris K, Aicart-Ramos C, Aramayo R, Montoya A, Kramer H, Rappsilber J, Torres-Rosell J, Moreno-Herrero F, Aragon L. Purified Smc5/6 Complex Exhibits DNA Substrate Recognition and Compaction. Mol Cell. 2020 Dec 17;80(6):1039–1054.e6. doi: 10.1016/j.molcel.2020.11.012 33301732 PMC7758880

[48] Serrano D, Cordero G, Kawamura R, Sverzhinsky A, Sarker M, Roy S, Malo C, Pascal JM, Marko JF, D’Amours D. The Smc5/6 Core Complex Is a Structure-Specific DNA Binding and Compacting Machine. Mol Cell. 2020 Dec 17;80(6):1025–1038.e5. doi: 10.1016/j.molcel.2020.11.011 33301731 PMC8224240

[49] Potts PR, Yu H. The SMC5/6 complex maintains telomere length in ALT cancer cells through SUMOylation of telomere-binding proteins. Nat Struct Mol Biol. 2007 Jul;14(7):581–90. doi: 10.1038/nsmb1259 17589526

[50] Min J, Wright WE, Shay JW. Alternative Lengthening of Telomeres Mediated by Mitotic DNA Synthesis Engages Break-Induced Replication Processes. Mol Cell Biol. 2017 Sep 26;37(20):e00226–17. doi: 10.1128/MCB.00226-17 28760773 PMC5615184

[51] Huttlin EL, Bruckner RJ, Navarrete-Perea J, Cannon JR, Baltier K, Gebreab F, Gygi MP, Thornock A, Zarraga G, Tam S, Szpyt J, Gassaway BM, Panov A, Parzen H, Fu S, Golbazi A, Maenpaa E, Stricker K, Guha Thakurta S, Zhang T, Rad R, Pan J, Nusinow DP, Paulo JA, Schweppe DK, Vaites LP, Harper JW, Gygi SP. Dual proteome-scale networks reveal cell-specific remodeling of the human interactome. Cell. 2021 May 27;184(11):3022–3040.e28. doi: 10.1016/j.cell.2021.04.011 33961781 PMC8165030

[52] Oughtred R, Rust J, Chang C, Breitkreutz BJ, Stark C, Willems A, Boucher L, Leung G, Kolas N, Zhang F, Dolma S, Coulombe-Huntington J, Chatr-Aryamontri A, Dolinski K, Tyers M. The BioGRID database: A comprehensive biomedical resource of curated protein, genetic, and chemical interactions. Protein Sci. 2021 Jan;30(1):187–200. doi: 10.1002/pro.3978 33070389 PMC7737760

[53] Gullberg M, Fredriksson S, Taussig M, Jarvius J, Gustafsdottir S, Landegren U. A sense of closeness: protein detection by proximity ligation. Curr Opin Biotechnol. 2003 Feb;14(1):82–6. doi: 10.1016/S0958-1669(02)00011-3 12566006

[54] McLaurin J, Trudel GC, Shaw IT, Antel JP, Cashman NR. A human glial hybrid cell line differentially expressing genes subserving oligodendrocyte and astrocyte phenotype. J Neurobiol. 1995 Feb;26(2):283–93. doi: 10.1002/neu.480260212 7707048

[55] Uhlén M, Fagerberg L, Hallström BM, Lindskog C, Oksvold P, Mardinoglu A, Sivertsson Å, Kampf C, Sjöstedt E, Asplund A, Olsson I, Edlund K, Lundberg E, Navani S, Szigyarto CA, Odeberg J, Djureinovic D, Takanen JO, Hober S, Alm T, Edqvist PH, Berling H, Tegel H, Mulder J, Rockberg J, Nilsson P, Schwenk JM, Hamsten M, von Feilitzen K, Forsberg M, Persson L, Johansson F, Zwahlen M, von Heijne G, Nielsen J, Pontén F. Proteomics. Tissue-based map of the human proteome. Science. 2015 Jan 23;347(6220):1260419. doi: 10.1126/science.1260419 25613900

[56] Kriventseva EV, Kuznetsov D, Tegenfeldt F, Manni M, Dias R, Simão FA, Zdobnov EM. OrthoDB v10: sampling the diversity of animal, plant, fungal, protist, bacterial and viral genomes for evolutionary and functional annotations of orthologs. Nucleic Acids Res. 2019 Jan 8;47(D1):D807–D811. doi: 10.1093/nar/gky1053 30395283 PMC6323947

[57] Granneman S, Gallagher JE, Vogelzangs J, Horstman W, van Venrooij WJ, Baserga SJ, Pruijn GJ. The human Imp3 and Imp4 proteins form a ternary complex with hMpp10, which only interacts with the U3 snoRNA in 60-80S ribonucleoprotein complexes. Nucleic Acids Res. 2003 Apr 1;31(7):1877–87. doi: 10.1093/nar/gkg300 12655004 PMC152815

[58] Thul PJ, Åkesson L, Wiking M, Mahdessian D, Geladaki A, Ait Blal H, Alm T, Asplund A, Björk L, Breckels LM, Bäckström A, Danielsson F, Fagerberg L, Fall J, Gatto L, Gnann C, Hober S, Hjelmare M, Johansson F, Lee S, Lindskog C, Mulder J, Mulvey CM, Nilsson P, Oksvold P, Rockberg J, Schutten R, Schwenk JM, Sivertsson Å, Sjöstedt E, Skogs M, Stadler C, Sullivan DP, Tegel H, Winsnes C, Zhang C, Zwahlen M, Mardinoglu A, Pontén F, von Feilitzen K, Lilley KS, Uhlén M, Lundberg E. A subcellular map of the human proteome. Science. 2017 May 26;356(6340):eaal3321. doi: 10.1126/science.aal3321 28495876

[59] Wyler E, Zimmermann M, Widmann B, Gstaiger M, Pfannstiel J, Kutay U, Zemp I. Tandem affinity purification combined with inducible shRNA expression as a tool to study the maturation of macromolecular assemblies. RNA. 2011 Jan;17(1):189–200. doi: 10.1261/rna.2325911 21097556 PMC3004060

[60] Faza MB, Chang Y, Occhipinti L, Kemmler S, Panse VG. Role of Mex67-Mtr2 in the nuclear export of 40S pre-ribosomes. PLoS Genet. 2012;8(8):e1002915. doi: 10.1371/journal.pgen.1002915 22956913 PMC3431309

[61] Smothers JF, Henikoff S. The HP1 chromo shadow domain binds a consensus peptide pentamer. Curr Biol. 2000 Jan 13;10(1):27–30. doi: 10.1016/S0960-9822(99)00260-2 10660299

[62] Liang J, Zhao H, Diplas BH, Liu S, Liu J, Wang D, Lu Y, Zhu Q, Wu J, Wang W, Yan H, Zeng YX, Wang X, Jiao Y. Genome-Wide CRISPR-Cas9 Screen Reveals Selective Vulnerability of ATRX-Mutant Cancers to WEE1 Inhibition. Cancer Res. 2020 Feb 1;80(3):510–523. doi: 10.1158/0008-5472.CAN-18-3374 31551363

[63] Rodriguez R, Müller S, Yeoman JA, Trentesaux C, Riou JF, Balasubramanian S. A novel small molecule that alters shelterin integrity and triggers a DNA-damage response at telomeres. J Am Chem Soc. 2008 Nov 26;130(47):15758–9. doi: 10.1021/ja805615w 18975896 PMC2746963

[64] Bechter OE, Zou Y, Walker W, Wright WE, Shay JW. Telomeric recombination in mismatch repair deficient human colon cancer cells after telomerase inhibition. Cancer Res. 2004 May 15;64(10):3444–51. doi: 10.1158/0008-5472.CAN-04-0323 15150096

[65] Garcia-Exposito L, Bournique E, Bergoglio V, Bose A, Barroso-Gonzalez J, Zhang S, Roncaioli JL, Lee M, Wallace CT, Watkins SC, Opresko PL, Hoffmann JS, O’Sullivan RJ. Proteomic Profiling Reveals a Specific Role for Translesion DNA Polymerase n in the Alternative Lengthening of Telomeres. Cell Rep. 2016 Nov 8;17(7):1858–1871. doi: 10.1016/j.celrep.2016.10.048 27829156 PMC5406014

[66] Déjardin J, Kingston RE. Purification of proteins associated with specific genomic Loci. Cell. 2009 Jan 9;136(1):175–86. doi: 10.1016/j.cell.2008.11.045 19135898 PMC3395431

[67] Grolimund L, Aeby E, Hamelin R, Armand F, Chiappe D, Moniatte M, Lingner J. A quantitative telomeric chromatin isolation protocol identifies different telomeric states. Nat Commun. 2013;4:2848. doi: 10.1038/ncomms3848 24270157

[68] Gao XD, Tu LC, Mir A, Rodriguez T, Ding Y, Leszyk J, Dekker J, Shaffer SA, Zhu LJ, Wolfe SA, Sontheimer EJ. C-BERST: defining subnuclear proteomic landscapes at genomic elements with dCas9-APEX2. Nat Methods. 2018 Jun;15(6):433–436. doi: 10.1038/s41592-018-0006-2 29735996 PMC6202229

[69] Zhou XZ, Lu KP. The Pin2/TRF1-interacting protein PinX1 is a potent telomerase inhibitor. Cell. 2001 Nov 2;107(3):347–59. doi: 10.1016/S0092-8674(01)00538-4 11701125

[70] Yoo JE, Oh BK, Park YN. Human PinX1 mediates TRF1 accumulation in nucleolus and enhances TRF1 binding to telomeres. J Mol Biol. 2009 May 22;388(5):928–40. doi: 10.1016/j.jmb.2009.02.051 19265708

[71] Jumper J, Evans R, Pritzel A, Green T, Figurnov M, Ronneberger O, Tunyasuvunakool K, Bates R, Žídek A, Potapenko A, Bridgland A, Meyer C, Kohl SAA, Ballard AJ, Cowie A, Romera-Paredes B, Nikolov S, Jain R, Adler J, Back T, Petersen S, Reiman D, Clancy E, Zielinski M, Steinegger M, Pacholska M, Berghammer T, Bodenstein S, Silver D, Vinyals O, Senior AW, Kavukcuoglu K, Kohli P, Hassabis D. Highly accurate protein structure prediction with AlphaFold. Nature. 2021 Aug;596(7873):583–589. doi: 10.1038/s41586-021-03819-2 34265844 PMC8371605

[72] Firat-Karalar EN, Rauniyar N, Yates JR 3rd, Stearns T. Proximity interactions among centrosome components identify regulators of centriole duplication. Curr Biol. 2014 Mar 17;24(6):664–70. doi: 10.1016/j.cub.2014.01.067 24613305 PMC4004176

[73] Van Itallie CM, Aponte A, Tietgens AJ, Gucek M, Fredriksson K, Anderson JM. The N and C termini of ZO-1 are surrounded by distinct proteins and functional protein networks. J Biol Chem. 2013 May 10;288(19):13775–88. doi: 10.1074/jbc.M113.466193 23553632 PMC3650414

[74] Tang J, Wu S, Liu H, Stratt R, Barak OG, Shiekhattar R, Picketts DJ, Yang X. A novel transcription regulatory complex containing death domain-associated protein and the ATR-X syndrome protein. J Biol Chem. 2004 May 7;279(19):20369–77. doi: 10.1074/jbc.M401321200 14990586

[75] Garrick D, Samara V, McDowell TL, Smith AJ, Dobbie L, Higgs DR, Gibbons RJ. A conserved truncated isoform of the ATR-X syndrome protein lacking the SWI/SNF-homology domain. Gene. 2004 Feb 4;326:23–34. doi: 10.1016/j.gene.2003.10.026 14729260

[76] McDowell TL, Gibbons RJ, Sutherland H, O’Rourke DM, Bickmore WA, Pombo A, Turley H, Gatter K, Picketts DJ, Buckle VJ, Chapman L, Rhodes D, Higgs DR. Localization of a putative transcriptional regulator (ATRX) at pericentromeric heterochromatin and the short arms of acrocentric chromosomes. Proc Natl Acad Sci U S A. 1999 Nov 23;96(24):13983–8. doi: 10.1073/pnas.96.24.13983 10570185 PMC24177

[77] Bérubé NG, Smeenk CA, Picketts DJ. Cell cycle-dependent phosphorylation of the ATRX protein correlates with changes in nuclear matrix and chromatin association. Hum Mol Genet. 2000 Mar 1;9(4):539–47. doi: 10.1093/hmg/9.4.539 10699177

[78] Ratnakumar K, Duarte LF, LeRoy G, Hasson D, Smeets D, Vardabasso C, Bönisch C, Zeng T, Xiang B, Zhang DY, Li H, Wang X, Hake SB, Schermelleh L, Garcia BA, Bernstein E. ATRX-mediated chromatin association of histone variant macroH2A1 regulates *α*-globin expression. Genes Dev. 2012 Mar 1;26(5):433–8. doi: 10.1101/gad.179416.111 22391447 PMC3305981

[79] Sarma K, Cifuentes-Rojas C, Ergun A, Del Rosario A, Jeon Y, White F, Sadreyev R, Lee JT. ATRX Directs Binding of PRC2 to Xist RNA and Polycomb Targets. Cell. 2014 Nov 20;159(5):1228. doi: 10.1016/j.cell.2014.11.010 28898627

[80] Nan X, Hou J, Maclean A, Nasir J, Lafuente MJ, Shu X, Kriaucionis S, Bird A. Interaction between chromatin proteins MECP2 and ATRX is disrupted by mutations that cause inherited mental retardation. Proc Natl Acad Sci U S A. 2007 Feb 20;104(8):2709–14. doi: 10.1073/pnas.0608056104 17296936 PMC1796997

[81] Kernohan KD, Jiang Y, Tremblay DC, Bonvissuto AC, Eubanks JH, Mann MR, Bérubé NG. ATRX partners with cohesin and MeCP2 and contributes to developmental silencing of imprinted genes in the brain. Dev Cell. 2010 Feb 16;18(2):191–202. doi: 10.1016/j.devcel.2009.12.017 20159591

[82] Gibbons RJ, McDowell TL, Raman S, O’Rourke DM, Garrick D, Ayyub H, Higgs DR. Mutations in ATRX, encoding a SWI/SNF-like protein, cause diverse changes in the pattern of DNA methylation. Nat Genet. 2000 Apr;24(4):368–71. doi: 10.1038/74191 10742099

[83] Law MJ, Lower KM, Voon HP, Hughes JR, Garrick D, Viprakasit V, Mitson M, De Gobbi M, Marra M, Morris A, Abbott A, Wilder SP, Taylor S, Santos GM, Cross J, Ayyub H, Jones S, Ragoussis J, Rhodes D, Dunham I, Higgs DR, Gibbons RJ. ATR-X syndrome protein targets tandem repeats and influences allele-specific expression in a size-dependent manner. Cell. 2010 Oct 29;143(3):367–78. doi: 10.1016/j.cell.2010.09.023 21029860

[84] Duc C, Benoit M, Détourné G, Simon L, Poulet A, Jung M, Veluchamy A, Latrasse D, Le Goff S, Cotterell S, Tatout C, Benhamed M, Probst AV. Arabidopsis ATRX Modulates H3.3 Occupancy and Fine-Tunes Gene Expression. Plant Cell. 2017 Jul;29(7):1773–1793. doi: 10.1105/tpc.16.00877 28684426 PMC5559740

[85] Udugama M, Sanij E, Voon HPJ, Son J, Hii L, Henson JD, Chan FL, Chang FTM, Liu Y, Pearson RB, Kalitsis P, Mann JR, Collas P, Hannan RD, Wong LH. Ribosomal DNA copy loss and repeat instability in ATRX-mutated cancers. Proc Natl Acad Sci U S A. 2018 May 1;115(18):4737–4742. doi: 10.1073/pnas.1720391115 29669917 PMC5939086

[86] Götz S, Pandey S, Bartsch S, Juranek S, Paeschke K. A Novel G-Quadruplex Binding Protein in Yeast-Slx9. Molecules. 2019 May 7;24(9):1774. doi: 10.3390/molecules24091774 31067825 PMC6539110

[87] Cosnier B, Kwapisz M, Hatin I, Namy O, Hermann-Le Denmat S, Morillon A, Rousset JP, Fabret C. A viable hypomorphic allele of the essential IMP3 gene reveals novel protein functions in Saccharomyces cerevisiae. PLoS One. 2011 Apr 29;6(4):e19500. doi: 10.1371/journal.pone.0019500 21559332 PMC3084874

[88] Cao Q, Wang X, Zhao M, Yang R, Malik R, Qiao Y, Poliakov A, Yocum AK, Li Y, Chen W, Cao X, Jiang X, Dahiya A, Harris C, Feng FY, Kalantry S, Qin ZS, Dhanasekaran SM, Chinnaiyan AM. The central role of EED in the orchestration of polycomb group complexes. Nat Commun. 2014;5:3127. doi: 10.1038/ncomms4127 24457600 PMC4073494

[89] Huttlin EL, Bruckner RJ, Paulo JA, Cannon JR, Ting L, Baltier K, Colby G, Gebreab F, Gygi MP, Parzen H, Szpyt J, Tam S, Zarraga G, Pontano-Vaites L, Swarup S, White AE, Schweppe DK, Rad R, Erickson BK, Obar RA, Guruharsha KG, Li K, Artavanis-Tsakonas S, Gygi SP, Harper JW. Architecture of the human interactome defines protein communities and disease networks. Nature. 2017 May 25;545(7655):505–509. doi: 10.1038/nature22366 28514442 PMC5531611

[90] Chudnovsky Y, Kim D, Zheng S, Whyte WA, Bansal M, Bray MA, Gopal S, Theisen MA, Bilodeau S, Thiru P, Muffat J, Yilmaz OH, Mitalipova M, Woolard K, Lee J, Nishimura R, Sakata N, Fine HA, Carpenter AE, Silver SJ, Verhaak RG, Califano A, Young RA, Ligon KL, Mellinghoff IK, Root DE, Sabatini DM, Hahn WC, Chheda MG. ZFHX4 interacts with the NuRD core member CHD4 and regulates the glioblastoma tumor-initiating cell state. Cell Rep. 2014 Jan 30;6(2):313–24. doi: 10.1016/j.celrep.2013.12.032 24440720 PMC4041390

[91] Happ H, Schilter KF, Weh E, Reis LM, Semina EV. 8q21.11 microdeletion in two patients with syndromic peters anomaly. Am J Med Genet A. 2016 Sep;170(9):2471–5. doi: 10.1002/ajmg.a.37840 27378168 PMC5119633

[92] Adhikari S, Thakur N, Shrestha U, Shrestha MK, Manshrestha M, Thapa B, Poudel M, Kunwar A. Genetic analysis of children with congenital ocular anomalies in three ecological regions of Nepal: a phase II of Nepal pediatric ocular diseases study. BMC Med Genet. 2020 Sep 22;21(1):185. doi: 10.1186/s12881-020-01116-9 32962661 PMC7510079

[93] Palomares M, Delicado A, Mansilla E, de Torres ML, Vallespín E, Fernandez L, Martinez-Glez V, García-Miñaur S, Nevado J, Simarro FS, Ruiz-Perez VL, Lynch SA, Sharkey FH, Thuresson AC, Annerén G, Belligni EF, Martínez-Fernández ML, Bermejo E, Nowakowska B, Kutkowska-Kazmierczak A, Bocian E, Obersztyn E, Martínez-Frías ML, Hennekam RC, Lapunzina P. Characterization of a 8q21.11 microdeletion syndrome associated with intellectual disability and a recognizable phenotype. Am J Hum Genet. 2011 Aug 12;89(2):295–301. doi: 10.1016/j.ajhg.2011.06.012 21802062 PMC3155189

[94] Kwon MS, Lee JJ, Min J, Hwang K, Park SG, Kim EH, Kim BC, Bhak J, Lee H. Brca2 abrogation engages with the alternative lengthening of telomeres via break-induced replication. FEBS J. 2019 May;286(10):1841–1858. doi: 10.1111/febs.14796 30811824

[95] Dilley RL, Verma P, Cho NW, Winters HD, Wondisford AR, Greenberg RA. Break-induced telomere synthesis underlies alternative telomere maintenance. Nature. 2016 Nov 3;539(7627):54–58. doi: 10.1038/nature20099 27760120 PMC5384111

[96] Tashiro S, Handa T, Matsuda A, Ban T, Takigawa T, Miyasato K, Ishii K, Kugou K, Ohta K, Hiraoka Y, Masukata H, Kanoh J. Shugoshin forms a specialized chromatin domain at subtelomeres that regulates transcription and replication timing. Nat Commun. 2016 Jan 25;7:10393. doi: 10.1038/ncomms10393 26804021 PMC4737732

[97] Ran FA, Hsu PD, Wright J, Agarwala V, Scott DA, Zhang F. Genome engineering using the CRISPR-Cas9 system. Nat Protoc. 2013 Nov;8(11):2281–2308. doi: 10.1038/nprot.2013.143 24157548 PMC3969860

[98] Lambert JP, Tucholska M, Go C, Knight JD, Gingras AC. Proximity biotinylation and affinity purification are complementary approaches for the interactome mapping of chromatin-associated protein complexes. J Proteomics. 2015 Apr 6;118:81–94. doi: 10.1016/j.jprot.2014.09.011 25281560 PMC4383713

[99] Liu G, Knight JD, Zhang JP, Tsou CC, Wang J, Lambert JP, Larsen B, Tyers M, Raught B, Bandeira N, Nesvizhskii AI, Choi H, Gingras AC. Data Independent Acquisition analysis in ProHits 4.0. J Proteomics. 2016 Oct 21;149:64–68. doi: 10.1016/j.jprot.2016.04.042 27132685 PMC5079801

[100] Perkins DN, Pappin DJ, Creasy DM, Cottrell JS. Probability-based protein identification by searching sequence databases using mass spectrometry data. Electrophoresis. 1999 Dec;20(18):3551–67. doi: 10.1002/(SICI)1522-2683(19991201)20:18<3551::AID-ELPS3551>3.0.CO;2-2 10612281

[101] Eng JK, Jahan TA, Hoopmann MR. Comet: an open-source MS/MS sequence database search tool. Proteomics. 2013 Jan;13(1):22–4. doi: 10.1002/pmic.201200439 23148064

[102] Shteynberg D, Deutsch EW, Lam H, Eng JK, Sun Z, Tasman N, Mendoza L, Moritz RL, Aebersold R, Nesvizhskii AI. iProphet: multi-level integrative analysis of shotgun proteomic data improves peptide and protein identification rates and error estimates. Mol Cell Proteomics. 2011 Dec;10(12):M111.007690. doi: 10.1074/mcp.M111.007690 21876204 PMC3237071

[103] Teo G, Liu G, Zhang J, Nesvizhskii AI, Gingras AC, Choi H. SAINTexpress: improvements and additional features in Significance Analysis of INTeractome software. J Proteomics. 2014 Apr 4;100:37–43. doi: 10.1016/j.jprot.2013.10.023 24513533 PMC4102138

[104] Knight JDR, Choi H, Gupta GD, Pelletier L, Raught B, Nesvizhskii AI, Gingras AC. ProHits-viz: a suite of web tools for visualizing interaction proteomics data. Nat Methods. 2017 Jun 29;14(7):645–646. doi: 10.1038/nmeth.4330 28661499 PMC5831326

[105] Raudvere U, Kolberg L, Kuzmin I, Arak T, Adler P, Peterson H, Vilo J. g:Profiler: a web server for functional enrichment analysis and conversions of gene lists (2019 update). Nucleic Acids Res. 2019 Jul 2;47(W1):W191–W198. doi: 10.1093/nar/gkz369 31066453 PMC6602461

[106] McQuin C, Goodman A, Chernyshev V, Kamentsky L, Cimini BA, Karhohs KW, Doan M, Ding L, Rafelski SM, Thirstrup D, Wiegraebe W, Singh S, Becker T, Caicedo JC, Carpenter AE. CellProfiler 3.0: Next-generation image processing for biology. PLoS Biol. 2018 Jul 3;16(7):e2005970. doi: 10.1371/journal.pbio.2005970 29969450 PMC6029841

[107] Abramoff M.D., Magalhaes P.J., Ram S.J. Image Processing with ImageJ Biophotonics International 2004; 7(11):36–42. 15084075

[108] Campos EI, Smits AH, Kang YH, Landry S, Escobar TM, Nayak S, Ueberheide BM, Durocher D, Vermeulen M, Hurwitz J, Reinberg D. Analysis of the Histone H3.1 Interactome: A Suitable Chaperone for the Right Event. Mol Cell. 2015 Nov 19;60(4):697–709. doi: 10.1016/j.molcel.2015.08.005 26527279 PMC4656108

[109] Williams ES, Bailey SM. Chromosome orientation fluorescence in situ hybridization (CO-FISH). Cold Spring Harb Protoc. 2009 Aug;2009(8):pdb.prot5269. doi: 10.1101/pdb.prot5269 20147245

[110] Mistry J, Chuguransky S, Williams L, Qureshi M, Salazar GA, Sonnhammer ELL, Tosatto SCE, Paladin L, Raj S, Richardson LJ, Finn RD, Bateman A. Pfam: The protein families database in 2021. Nucleic Acids Res. 2021 Jan 8;49(D1):D412–D419. doi: 10.1093/nar/gkaa913 33125078 PMC7779014

[111] Sigrist CJ, de Castro E, Cerutti L, Cuche BA, Hulo N, Bridge A, Bougueleret L, Xenarios I. New and continuing developments at PROSITE. Nucleic Acids Res. 2013 Jan;41(Database issue):D344–7. doi: 10.1093/nar/gks1067 23161676 PMC3531220

[112] Papadopoulos JS, Agarwala R. COBALT: constraint-based alignment tool for multiple protein sequences. Bioinformatics. 2007 May 1;23(9):1073–9. doi: 10.1093/bioinformatics/btm076 17332019

[113] Crooks GE, Hon G, Chandonia JM, Brenner SE. WebLogo: a sequence logo generator. Genome Res. 2004 Jun;14(6):1188–90. doi: 10.1101/gr.849004 15173120 PMC419797

