# Supplementary figures and images for: ATRX proximal protein associations boast roles beyond histone deposition
Source: PLoS Genet. 2021 Nov 15;17(11):e1009909. doi: 10.1371/journal.pgen.1009909 (PMC8629390; doi:10.1371/journal.pgen.1009909)

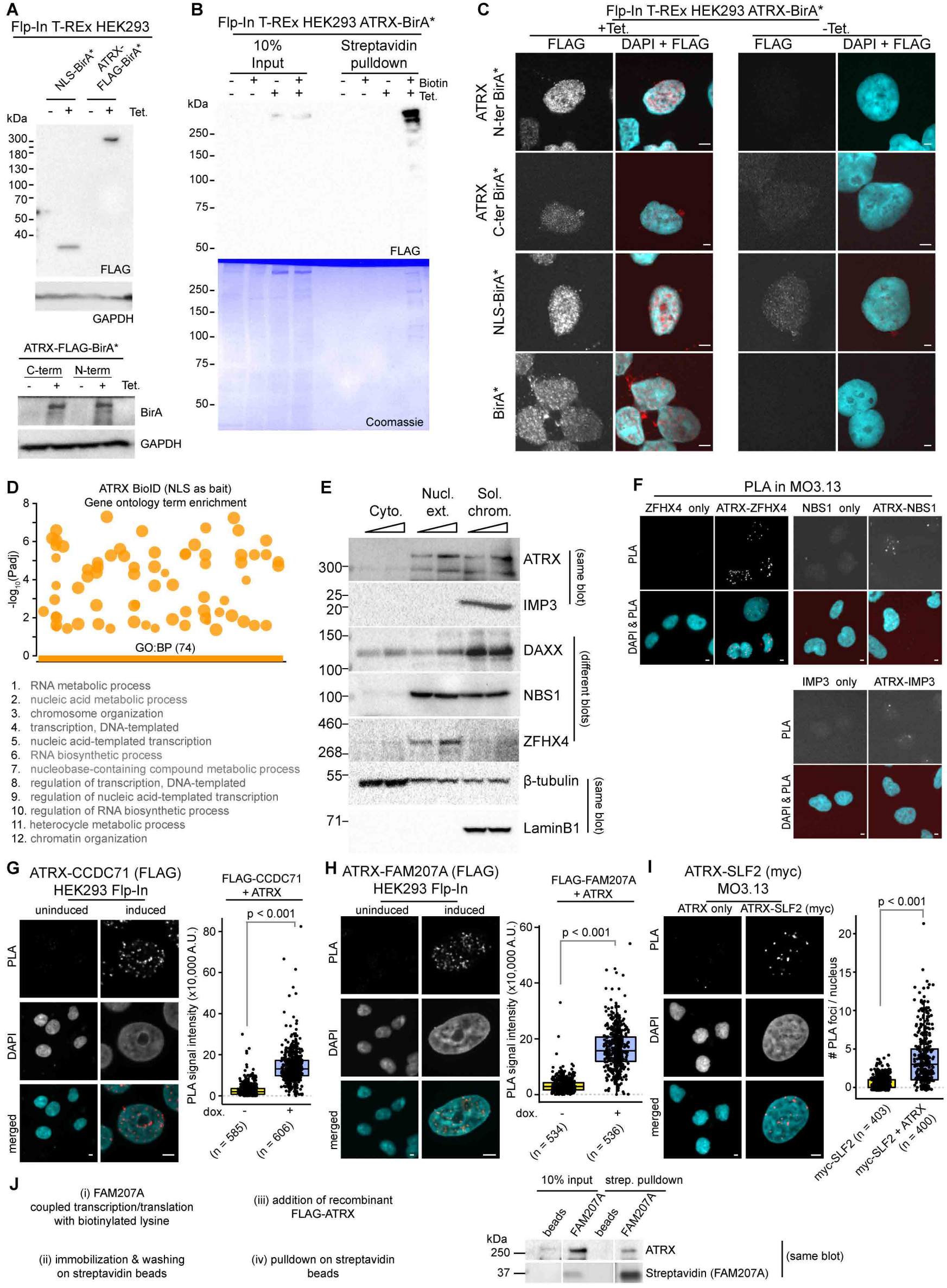

Supplement: S1 Fig — A: Western blot exemplifying stable integration and expression of FLAG-tagged BirA* protein fusions in inducible HEK293 Flp-In T-REx cells (top), and similar expression levels of N- and C-terminally-tagged ATRX (bottom). B: Western blot (top) and Coomassie-staining (bottom) of biotinylated proteins captured on streptavidin beads. C: Example of immunofluorescent labeling demonstrating nuclear targeting of the ATRX-BirA* fusion constructs. D: Gene ontology analysis of ATRX-associating proteins. E: Western blot showing the subcellular distribution of ATRX-associating proteins in HeLa S3 cells. F: Examples of the proximity ligation assay (PLA) showing associations between endogenous proteins in MO3.13 cells. G-H: PLAs showing associations between endogenous ATRX and exogenous FLAG-tagged CCDC71 (G) or FAM207A (H) in HEK293 Flp-In T-REx cells. I: PLAs showing an association between endogenous ATRX and myc-tagged SLF2. Data plots account for ∼100 nuclei in three independent experiments, with the total number of nuclei assessed in brackets. The p-values were obtained using a 2-sided Student’s t-test with unequal variance. J: In vitro interaction between recombinant FAM207A and ATRX. (PDF) [file pgen.1009909.s001.pdf]

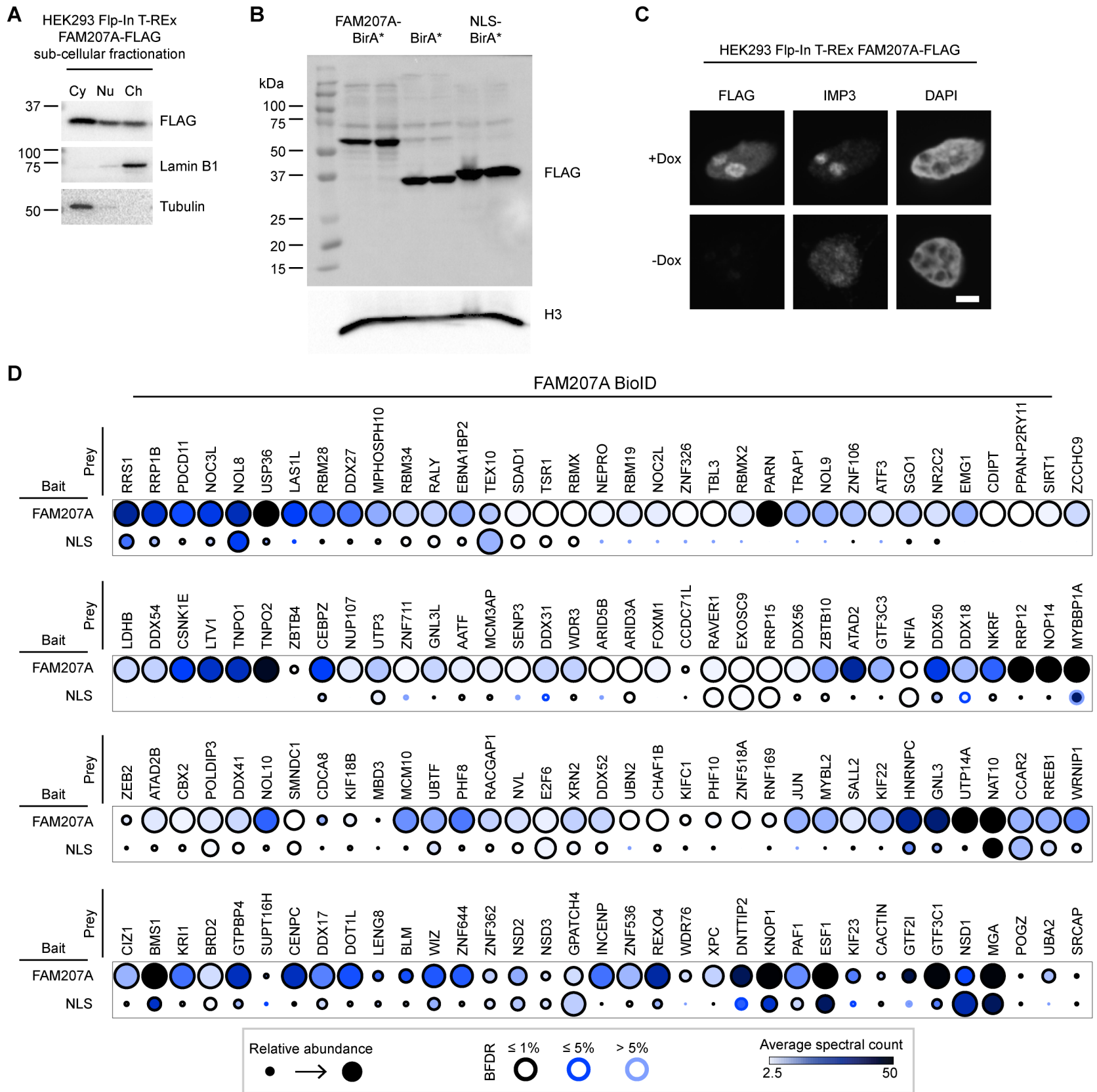

**E** Full blots for figure 2a

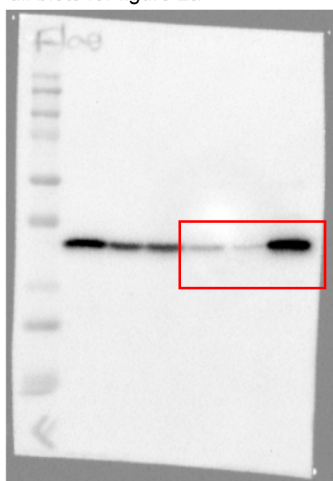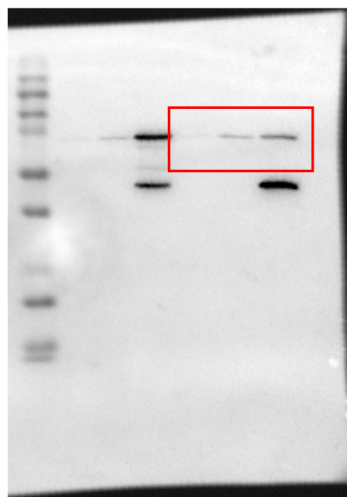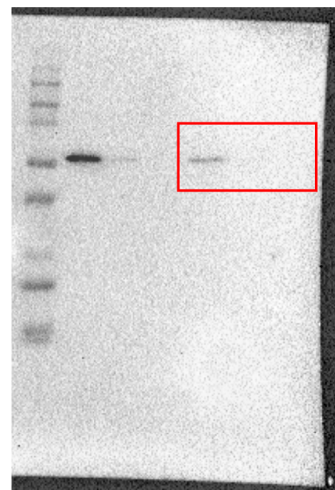

Supplement: S2 Fig — A: Subcellular fractionation of HEK293 Flp-In T-REx cells expressing FLAG-FAM207A. Cy—cytoplasm; Nu—nucleus; Ch—chromatin. B: Western analysis of BirA* constructs used for the FAM207A BioID experiment. Duplicate (induced) lanes are shown. C: Immunolabeling experiment showing co-localization of FAM207A and the IMP3 nucleolar protein. Scale bar = 4μm. D: Dot plot showing prey proteins identified with FAM207A-BirA* that were enriched over endogenous biotinylation (untransfected), unspecific pan-cellular biotinylation (BirA*), and unspecific nuclear biotinylation (NLS-BirA*; BFDR ≤ 5%, SAINT [103]). Data represent two biological replicates. Proteins in boldface remained statistically enriched when the nuclear localization signal (NLS)-BirA* control was used to further filter the FAM207A BioID data. E: Full western blots for Fig 2A. The red boxes indicate the areas shown in the main figure. (PDF) [file pgen.1009909.s002.pdf]

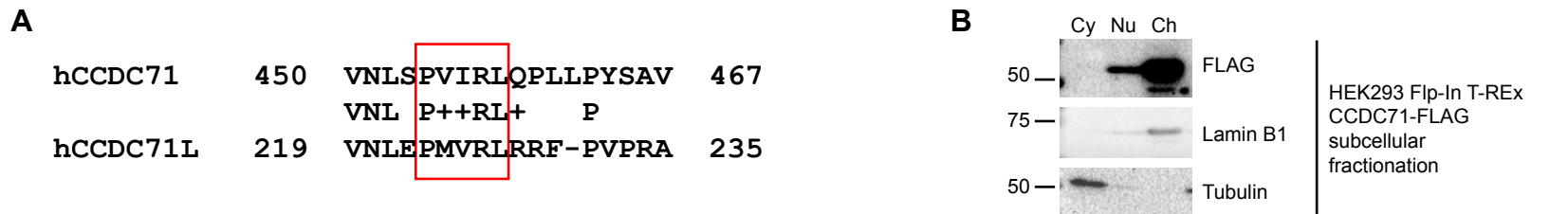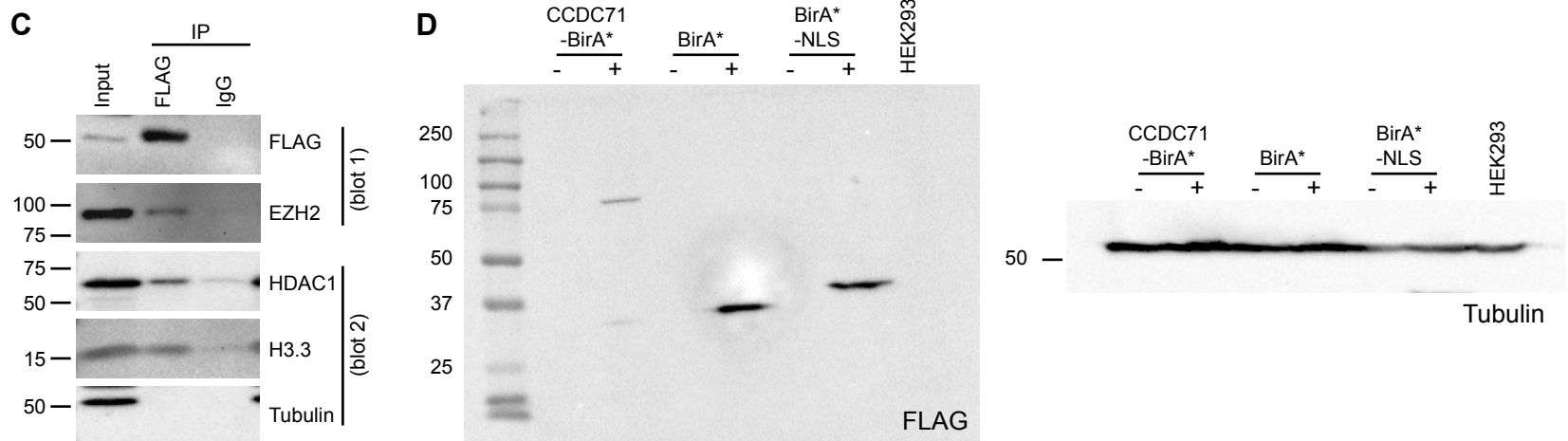

**F** Full blots for figure 3d

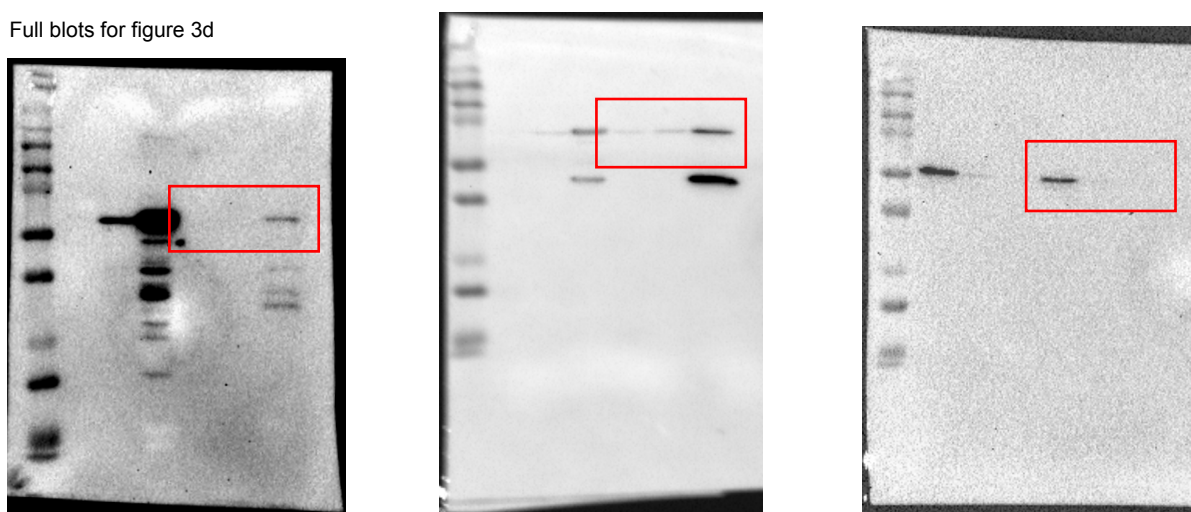

Supplement: S3 Fig — A: PxVxL motif in human CCDC71L. B: Subcellular fractionation of HEK293 Flp-In T-REx cells expressing FLAG-CCDC71. Cy—cytoplasm; Nu—nucleus; Ch—chromatin. C: CCDC71 immunoprecipitation and verification of associations reported in BioGrid [52]. D: Western analysis of CCDC71 and control BirA* constructs. E: Dot plot showing prey proteins identified with CCDC71-BirA* that were enriched over endogenous biotinylation (untransfected) and unspecific pan-cellular biotinylation (BirA*; BFDR ≤ 5%, SAINT [103]). Data represent two biological replicates. Proteins in boldface remained statistically enriched when the nuclear localization signal (NLS)-BirA* control was used to further filter the CCDC71 BioID data. F: Full western blots for Fig 3D. The red boxes indicate the areas shown in the main figure. (PDF) [file pgen.1009909.s003.pdf]

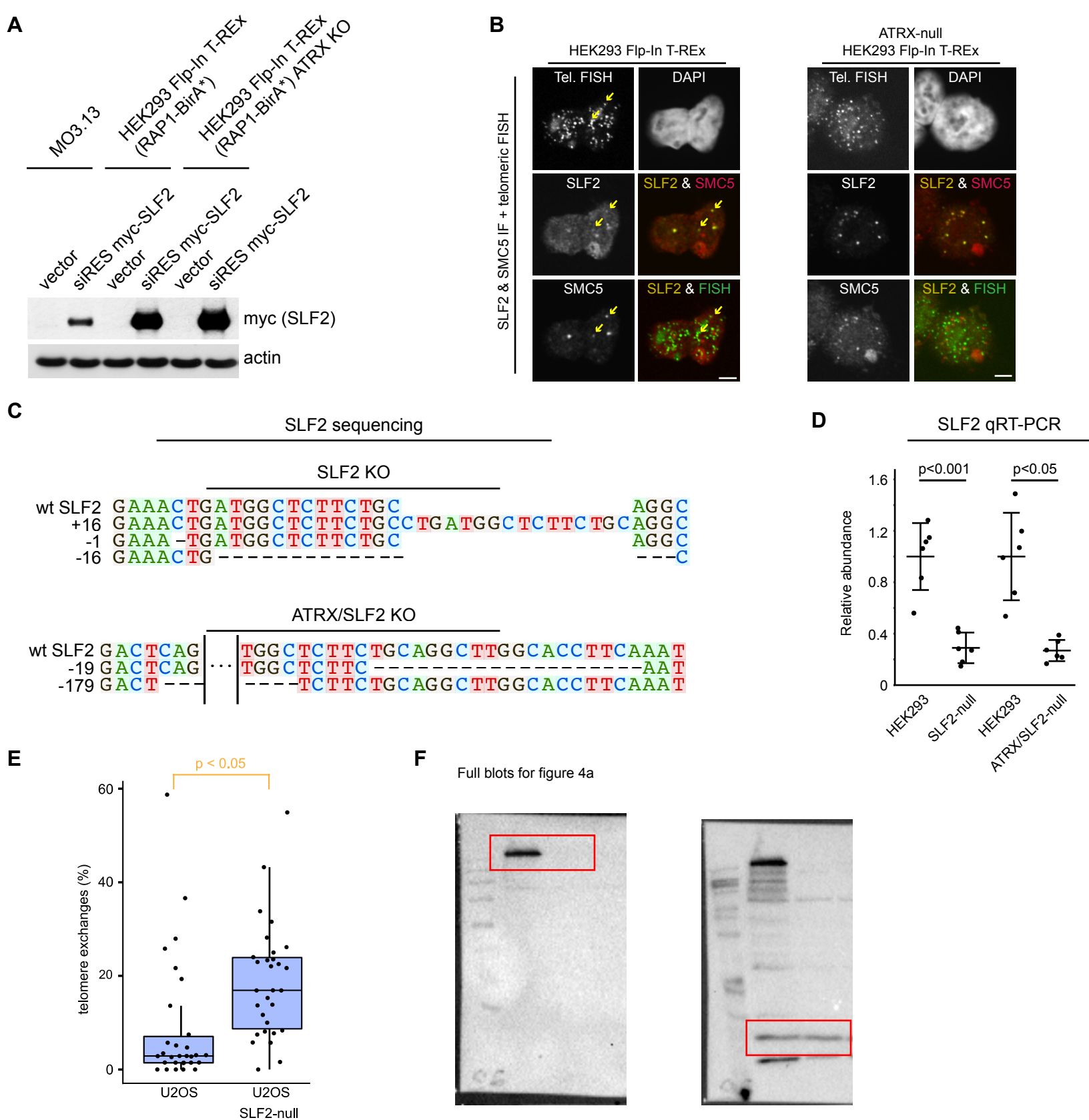

Supplement: S4 Fig — A: Western blot showing exogenous myc-SLF2 expression in lentivirus-infected HEK293 Flp-In T-REx (with inducible RAP1-BirA*, but the latter is not induced). B: Immunolabeling of myc-SLF2 (yellow) and SMC5 (red), and fluorescence in situ hybridization (IF-FISH) using a telomeric (green) probe in ATRX-expressing and -null HEK293 Flp-In cells. C: CRISPR-Cas9-mediated SLF2 gene disruptions in HEK293 Flp-In T-REx (RAP1-BirA*), detected by DNA sequencing, and matching qRT-PCR (D). E: Telomere exchange rates observed in U2OS cells. At least 30 mitotic spreads per condition were counted and the percent of telomeric exchanges plotted. F: Full western blots for Fig 4A. The red boxes indicate the areas shown in the main figure. (PDF) [file pgen.1009909.s004.pdf]

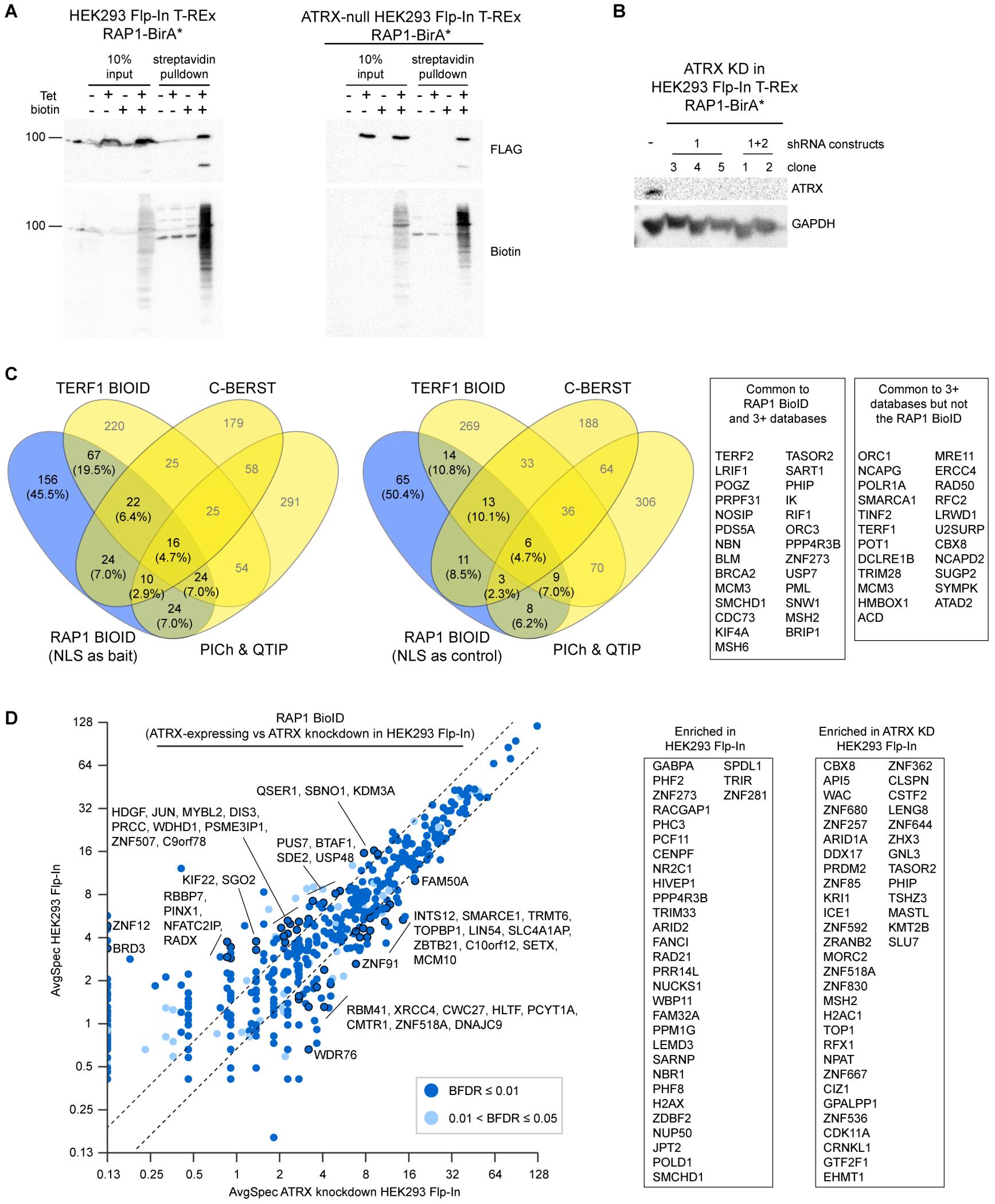

Supplement: S5 Fig — A: Western blot showing the induction of FLAG- and BirA*-tagged RAP1 in ATRX-expressing and -null HEK293 Flp-In T-REx cells. B: Western analysis of shRNA-mediated ATRX knockdown in HEK293 Flp-In T-REx cells expressing RAP1-BirA*. Doubly transfected cells (shRNA constructs 1 and 2) were used for the RAP1 BioID. C: Overlap between RAP1 BioID and TERF1 BioID [65], dCas9-APEX2 biotinylation at genomic elements by restricted spatial tagging (C-BERST) [68], quantitative telomeric chromatin isolation protocol (QTIP) [67], and proteomics of isolated chromatin segments (PICh) [66]. PICh and QTIP data were grouped to simplify the Venn diagrams. Data consider HEK293 Flp-In cells expressing ATRX (unperturbed). A list of proteins commonly identified in at least three different telomere proteomic screens is shown on the right. D: Fold change of prey proteins identified by RAP1 BioID (BFDR ≤ 5%, SAINT [103]) in ATRX KD vs. ATRX-expressing HEK293 Flp-In cells. Labeled proteins had ≥2.5 average spectra and ≥1.5-fold change between conditions. Proteins labeled in the scatter plot remained significant when NLS-BirA* was used as a control. Inset box represents proteins also identified with NLS-BirA* as a bait (less stringent). (PDF) [file pgen.1009909.s005.pdf]
